# Shaping the Physicochemical and Health-Promoting Properties of Carrot Snacks Produced by Microwave-Vacuum Drying with Preliminary Thermal and Enriching Treatment

**DOI:** 10.3390/molecules29215100

**Published:** 2024-10-29

**Authors:** Anna Ignaczak, Łukasz Woźniak, Agnieszka Salamon, Justyna Szczepańska-Stolarczyk, Urszula Trych, Małgorzata Chobot, Jolanta Kowalska, Hanna Kowalska

**Affiliations:** 1 Department of Food Engineering and Process Management, Institute of Food Sciences, Warsaw University of Life Sciences, 159c Nowoursynowska St., 02-776 Warsaw, Poland; malgorzata_chobot@sggw.edu.pl (M.C.); jolanta_kowalska@sggw.edu.pl (J.K.); 2 Department of Fruit and Vegetable Product Technology, Institute of Agricultural and Food Biotechnology-State Research Institute, 36 Rakowiecka St., 02-532 Warsaw, Poland; justyna.szczepanska@ibprs.pl (J.S.-S.); urszula.trych@ibprs.pl (U.T.); 3 Department of Food Safety and Chemical Analysis, Institute of Agricultural and Food Biotechnology-State Research Institute, 36 Rakowiecka St., 02-532 Warsaw, Poland; lukasz.wozniak@ibprs.pl; 4 Department of Grain Processing and Bakery, Institute of Agricultural and Food Biotechnology-State Research Institute, 36 Rakowiecka St., 02-532 Warsaw, Poland; agnieszka.salamon@ibprs.pl

**Keywords:** carrot snacks, osmotic enriching, drying methods, technological properties, carotenoids profile, phenolics, anthocyanins, vitamin C content, antioxidant activity, sugar profile

## Abstract

This study analyzed the effects of thermal pre-treatments such as convective drying (P-CD), water (BL_W), and microwave blanching (M_BL) and osmotic enrichment pre-treatments with juices from pomegranate (PG), chokeberry (CH), and sea buckthorn (SB) on microwave-vacuum-dried (MVD) carrot properties. Convective drying (CD) and freeze-drying (FD) were used as a comparative method. The dry matter content and water activity of MVD carrots were varied, but in many cases, the values were comparable to those of FD-dried carrots. Pre-enrichment in CH juice significantly reduced the values of the color parameters L*, a*, and b*, regardless of the drying method. The smallest changes were observed in microwave pre-blanching (M_BL). The lowest loss in carotenoid content was observed in CD-dried carrots (14–34 mg/100 g d.m.). Blanching and enrichment in SB juice allowed significant retention of these compounds. As a result of drying carrots, the total phenolic content (TPC) increased. Compared to the raw material, the TPC content in dried carrots increased 3–9 times. Drying using the FD and MVD methods gave a similar effect of increasing the TPC content, including a greater effect after enrichment in CH juice. The highest average antioxidant activity against the DPPH• and ABTS•+ radicals was recorded for FD-dried carrots (6.9 and 30.0 mg Trolox/g d.m.). SB juice contributed to a significant increase in the total vitamin C content, even by 89.1%, compared to raw carrots. Applying osmotic pre-enrichment in PG juice increased the sugar content in dried FD and CD samples by 37.4–49.9%, and in MVD by 21–59%.

## 1. Introduction

There is an increase in consumer awareness of the importance of human nutrition and the impact of food on the proper functioning of the body. Proper nutrition has a beneficial effect on many metabolic processes, including providing energy, growth, and regeneration and increasing the body’s resistance to diseases [1]. Understanding the need to compose a daily diet with a large amount of fruit and vegetables, which are a rich source of dietary fiber, minerals, and vitamins, causes food producers to develop new technologies enabling the production of high-value food, including snacks in the form of fresh or dried crunchy fruit and vegetables [2].

Due to their high moisture content (above 80%), fresh fruits and vegetables have a short shelf life. These raw materials are sensitive to environmental factors and susceptible to mechanical damage and therefore also to the development of harmful microflora [3,4]. Vegetable preservation, and consequently the production of vegetable snacks of different flavors, shapes, and consistencies, using appropriate drying methods with the use of various auxiliary procedures, such as osmotic enrichment in juices or blanching, can become an interesting alternative to the production of snacks of the desired sensory and health quality. The modern consumer expects such snacks. Therefore, in recent years, the market has seen increased demand for such products. At the beginning of the 1990s, the value of the European market for dried vegetables increased significantly, from USD 260 million to almost USD 1 billion [5]. The latest data show that the broadly understood global healthy snack market in 2021 amounted to USD 85.6 billion and was estimated to grow by 6.6% annually between 2022 and 2030. Hence, the market will reach USD 152.3 billion in 2030 [6]. It should be emphasized that the selection of production conditions and testing of the properties of fruit snacks with health-promoting properties and good sensory quality is the subject of many studies described in publications [3]. Fruit snacks are increasingly available on the market and are often consumed. There is a lack of research on vegetable snacks with health-promoting properties, both in terms of technological indicators and those concerning the properties of dried vegetables. The market is still dominated by snacks produced by frying, extrusion, or other methods, leading to the production of highly processed products often devoid of many native ingredients, with an unfavorable fatty acid profile, or with the addition of undesirable chemical compounds. 

Carrots are of great importance in the production of dried vegetable snacks. They are a leading garden vegetable from the *Apiaceae* family and one of the most valuable root vegetables in terms of nutritional value. At the same time, due to their high water content, they are a low-calorie vegetable (42 kcal/100 g) [7]. Carrots contain approximately 88% water, 1% protein, 7% carbohydrates, 0.2% fat, and 3% dietary fiber. Depending on the type of carotenoid and anthocyanin pigments accumulated in carrot roots, they take on a variety of colors. The most popular is the orange carrot root, which contains significant amounts of vitamins, including B1, B2, B6, PP, folic acid, and, above all, vitamin A, necessary for proper vision. It is also rich in minerals such as calcium, magnesium, potassium, phosphorus, and iron [8,9]. Carrots are one of the most important sources of carotenoids, especially β-carotene (approx. 8.3 mg/100 g), which has strong anti-cancer and antioxidant properties [10,11].

Drying is one of the most promising methods of producing vegetable snacks, which allows the reduction of the water content in the raw material to a level that ensures its microbiological stability, slowing down enzymatic transformations and ensuring an extension of the shelf life [12]. The selection of the drying method and conditions is important. Exposing the material to high temperature or long-term drying contributes to the loss of native, thermolabile, and nutritionally valuable components of the raw material [11,13], as well as high energy consumption and production costs [14]. Currently, solutions are being developed to shorten the drying time and improve the efficiency and quality of the dried product. Microwave-vacuum drying, in which the simultaneous action of microwaves and reduced pressure increases the speed of the process, is an increasingly promising method of producing vegetable snacks. Due to the intensive evaporation of moisture under the influence of microwaves and the reduction of the boiling point of water at reduced pressure, drying is not only more efficient but also protects the product from overheating and preserves ingredients sensitive to high temperature [15].

An important procedure in producing dried vegetables is pre-treatment, both thermal and enrichment. The main task of the blanching treatment of vegetables is to inactivate enzymes, such as peroxidase, polyphenol oxidase, and pectinmethylesterase, shaping the physical, sensory, and health-promoting properties of food and also microbiological quality [16,17]. This process also affects the efficiency of drying, freezing, and others. Blanching carrot root is particularly useful for shaping the sensory characteristics and health-promoting values of dried vegetables [11,18]. Food enrichment with various ingredients is currently a common topic of many scientific studies, as well as a branch of food processing. This results from the need to supplement the deficiencies of micro and macro elements, such as vitamins, minerals, and dietary fiber, which are responsible for various ailments and are symptoms of many people’s diseases [19]. One way to fortify food is biofortification, which involves agronomic practices, conventional plant breeding, or modern biotechnology [20]. A common food enrichment procedure is osmotic pre-treatment before drying [21,22] or freezing [23]. Its purpose, in addition to compensating for the loss of ingredients or introducing new ones into food, is to produce attractive vegetable snacks. Not from concentrate (NFC) or concentrated juices are currently used as an alternative to refined sugars or fructose-glucose syrups. The effect of enrichment with juice components or osmotic dehydration in concentrated juices allows for an effective increase in the amount of bioactive compounds [11,24,25]. Various enrichment techniques are used, such as vacuum enriching or the use of coatings with enriching ingredients that at the same time protect against the loss of native ingredients. Depending on the process conditions used, including the type of enrichment solution, its concentration, temperature, and duration, enrichment compounds can penetrate the porous material (plant tissue) or create a barrier for compounds to the outside with appropriate water-loss control [26,27]. Using pre-enrichment in juices affects the health-promoting as well as sensory attributes such as color, acidity, tartness, and sweetness [28].

This study aimed to assess the possibility of using pre-treatments such as convective drying, water, and microwave blanching and osmotic enrichment in NFC sea buckthorn, chokeberry, and pomegranate juices before microwave-vacuum drying with various pressure and microwave power parameters. To assess the impact of these factors, comparative drying of carrots using convection and freeze-drying methods was used. The scope of this work included an assessment of the effect of drying conditions on the physicochemical properties of dried carrots in terms of obtaining high-value vegetable snacks and technological methods enabling lower energy use compared to traditional methods.

## 2. Results and Discussion

### 2.1. Influence of Pre-Treatment, Drying Method, and Microwave-Vacuum-Drying Parameters on Physical Properties of Dried Carrot (The Technological Parameters)

#### 2.1.1. Dry Matter Content and Water Activity of Fresh and Dried Carrot

The dry matter content in fresh carrots was about 14.4% (Table 1). According to literature data [29,30], in carrots, it ranges from 10 to 12%. Differences may result from, among others, carrot variety, degree of maturity, cultivation method, and storage method. In dried carrot samples, dry matter content ranged from 76.0 to 99.6%. A significant effect of the drying method and type of pre-treatment on the value of this indicator in dried carrots was found. A significant interdependent effect of pressure and microwave power of microwave-vacuum drying was also demonstrated. The highest dry matter content (95.3 to 99.6%) was recorded in freeze-dried samples. Among the freeze-dried products, the lowest values were observed in the samples pre-enriched with pomegranate juice (95.3%) and the highest in the water-blanched carrot (99.6%). Convective (88.7%) and microwave-vacuum-dried (90.4%) products were characterized by significantly lower average dry matter content than the freeze-dried products. In the convective dried products, slightly lower dry matter content was noted in the carrot samples pre-enriched with pomegranate juice (85.6%) and higher after initial microwave blanching (91.8%). 

In comparing microwave-vacuum-dried carrot samples, the dry matter content ranged from 76.0 to 98.6%. Pre-treatment, consisting of enriching carrots with components of chokeberry (89.7%) and pomegranate (91.3%) juices and microwave blanching (91.4%), did not significantly affect the parameter value compared to samples without (86.3%). Significantly higher values were noted in carrots enriched with sea buckthorn juice (92.8%), convectively dried (93.0%), and blanched in water (93.4%).

The applied parameters of pressure and microwave power significantly influenced the dry matter content in the obtained dried carrot. The average dry matter content in dried products by using a microwave power of 250 W and a pressure of 3.5 kPa was characterized by a significantly higher value, at the level of 92.7%, compared to the dried products obtained at a pressure of 6.5 kPa and amounted to about 87.7%. At a microwave power of 400 W, the applied pressure did not significantly influence the dry matter content. The average values in MVD dried samples were 89.6% at 3.5 kPa and 91.4% at 6.5 kPa.

The water activity of dried carrots varied from 0.070 to 0.565 (Table 1). Therefore, regardless of the drying method and conditions of the MVD method and pre-treatment, the dried carrot samples showed high microbiological stability. The required threshold of 0.6 was not exceeded. It should be noted that the drying method, which has a significant effect on water activity, is important regarding the quality of the dried products. According to the rule, the lower the water activity, the more stable the product. It was assumed that the highest stability is characterized by products whose water activity is 0.070–0.350 [31]. A statistically significant effect of the carrot drying method and the interaction of microwave-vacuum-drying parameters, i.e., pressure and microwave power, was demonstrated. However, no significant effect of the type of pre-treatment on the value of the discussed indicator was found, but certain trends were observed. The lowest water activity (0.070–0.135) was characteristic of freeze-dried samples. Significantly higher values were obtained for convective-dried samples (0.310–0.442); the highest was for carrot samples initially osmotically enriched with pomegranate juice and the lowest was for samples enriched with sea buckthorn juice. Significantly higher, in comparison to freeze-dried samples, and the most diverse values of water activity were characteristic of microwave-vacuum-dried carrots (0.202–0.565). Similarly to the case of dry matter content, only the interaction of pressure and microwave power parameters during microwave-vacuum drying significantly affected the level of water activity in dried carrot samples. A significantly lower average water activity of 0.360 was characteristic for the dried plants obtained at the pressure and microwave power of 3.5 kPa/250 W and 6.5 kPa/400 W. A separate homogeneous group (B’) was formed by the dried plants obtained at the pressure of 3.5 kPa/400 W and 6.5 kPa/250 W with an average water activity of 0.450. In samples dried by MVD at a pressure of 6.5 kPa and microwave power of 400 W after enrichment in PG juice, convective drying, and without pre-treatment, water activity was relatively lower (0.213–0.283). At a pressure of 3.5 kPa and microwave power of 250 and 400 W, similar values (0.202–0.254) were found in samples dried after enrichment with SB juice. In most of the other samples dried by this method, the values were higher. This may be mainly due to difficulties related to non-uniform heating of the material or even local burning [32]. 

The wide range of values may result from the difficulties in selecting drying parameters for this method due to many component parameters, including pressure, microwave power, dielectric properties, and structure and composition of food, which determine the appropriate degree of drying of the material while maintaining its visual acceptability [33]. At the same time, both through convective pre-drying and blanching, the moisture content of dried carrots, described by the carrot dry matter content change index, was appropriately reduced so that drying by the MVD method could be carried out. Considering the previous and current studies, it can be stated that the selection of MVD parameters (microwave power and operation time, use of breaks, and vacuum size) is more important than the type of pre-treatment. Moreover, blanching is much more advantageous than pre-drying, because it is a very short treatment. However, the required humidity reduction before MVD is probably unnecessary in this case due to various physicochemical changes, including changes in the structure of plant tissue that facilitate drying.

#### 2.1.2. Mass Loss of Dried Carrot

As a result of drying carrots, a significant mass loss occurred (Table 1), independent of process conditions. The value of this indicator ranged from 82.1 to 94.2%. No statistically significant effect of the drying method and the correlation of pressure and microwave power used during microwave-vacuum drying of dried carrots was found. Only specific trends were observed. The average values of mass loss from the lowest to the highest value in dried carrots, depending on the drying method, were as follows: freeze-drying (FD; 85.8%) ⇒ microwave-vacuum method (MVD; 87.5%) ⇒ convection (CD; 88.1%). The type of pre-treatment used had a significant effect on the mass loss in the tested dried carrots. Pre-enrichment of carrots with components of chokeberry (CH) and pomegranate (PG) juices, 84.1–84.4%, resulted in significantly lower mass loss compared to control samples (88.1%), enriched with sea buckthorn juice (SB) and blanching, both microwave (M_BL) and water (W_BL), (88.5–89.7%). Significantly lower mass losses in the case of enrichment of carrots with components of CH and PG juices can be explained by the partial removal of water from carrot tissue with simultaneous penetration of NFC juice components during osmotic enrichment. Therefore, mass exchange during drying was lower. Similar findings were shown in earlier studies by Ignaczak et al. [11] on pre-blanching and osmotic dehydration of carrots in apple juice concentrate.

The final mass loss of carrot samples after drying could be influenced by the mass loss obtained after each of the applied pre-treatments, i.e., blanching (water, W_BL, and microwave, M_BL), enrichment in chokeberry (CH), pomegranate (PG), and sea buckthorn (SB) juices, as well as 30-minute convective drying (P-CD). The type of pre-treatment had a significant effect on the mass loss determined immediately after the treatment (Table 1). Drying P-CD resulted in 50.0–54.4% mass loss, significantly greater than in the remaining samples before CD and FD drying. Considering MVD, when convective drying was used in the next stage (after blanching and enrichment), despite the lack of significant differences, a tendency to obtain a similar mass loss was observed only in samples enriched in CH. In the remaining samples, the mass loss was even 24% greater. It was expected that the mass loss value would be a valuable technological indicator allowing to determine the moisture content of samples before MVD. It is difficult to connect the values of this indicator after pre-treatment with the final values after drying, regardless of the method used. However, it is an indication and justification for undertaking previously planned studies on changes in the microstructure and water status in dried carrot samples.

#### 2.1.3. Color Parameters of Dried Carrot of Fresh and Dried Carrot

**Figure 1:**
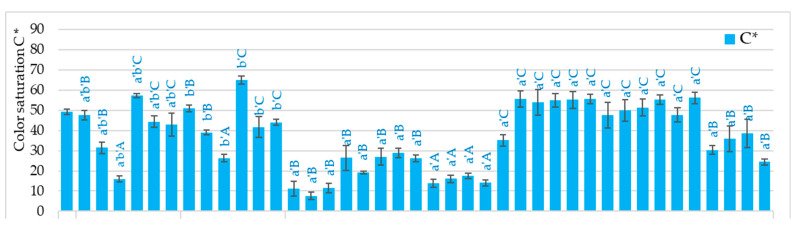

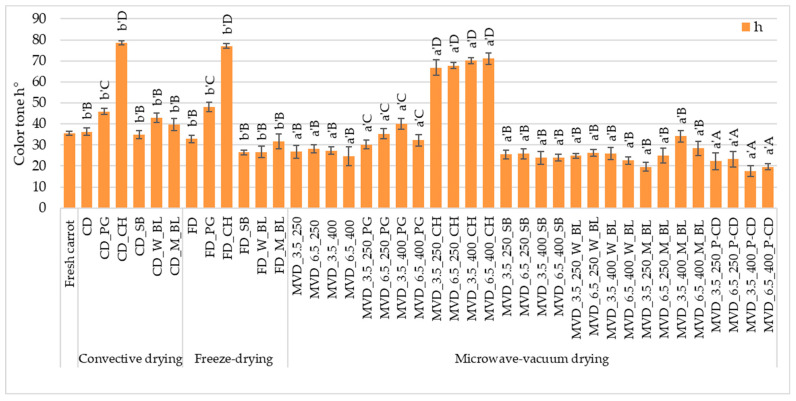
The effect of the type of osmotic enriching agent (NFC juice) and convective drying as pre-treatment, drying method, and microwave-vacuum-drying parameters on values of C* and h color parameters of dried carrots. Different letters a’, b’ (type of drying method); A, B, C, D (type of pre-treatment)—homogeneous groups show the statistical difference at *p* ≤ 0.05. When all the data were in one homogenous group (*p* > 0.05), a’, A letters were omitted. Code designations of dried samples are as in the caption of Table 1.

The fresh carrot was characterized by a lightness color (L*) of about 65.4, about 25.6 redness (a*), and about 36.8 yellow of approximately 36.8 (b*), and the intensity C* and color tone h* were 50.1 and 32.9, respectively (Tables S1 and S2, Figure 1). The type of drying method, microwave-vacuum-drying parameters and osmotic agent (NFC juice), and convective drying as pre-treatments resulted in obtaining a color significantly different from the color of the raw material (Table 2). 

**Figure 2:**
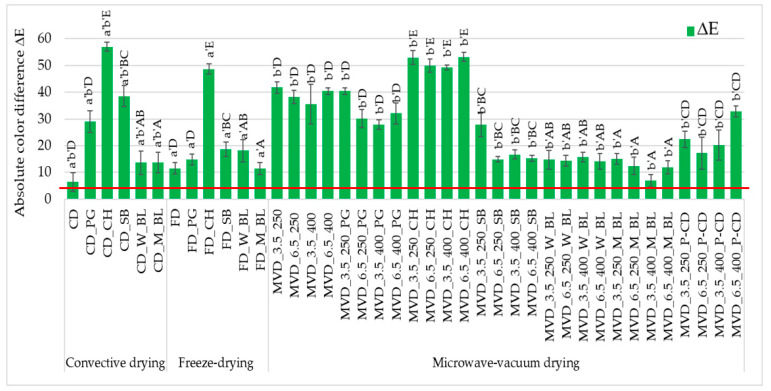
The effect of the type of osmotic pre-treatment (NFC juice) or thermal pre-treatment, drying method, and microwave-vacuum-drying parameters on the absolute color difference (ΔE) of dried carrot samples. Different letters a’, b’ (type of drying method); A, B, C, D, E (type of pre-treatment)—homogeneous groups show the statistical difference at *p* ≤ 0.05. When all the data were in one homogenous group (*p* > 0.05), a’, A letters were omitted. Code designations of dried samples are as in the caption of Table 1. The red line marks the limit of ΔE values above which color differences are visible to the average observer.

The absolute color difference values of all dried carrot samples were high and noticeable to an average observer (Figure 2). The smallest absolute color difference (approx. 6.4) of dried carrot samples concerned convection-dried samples. In contrast, the highest one was convectively dried samples after initial osmotic enrichment in chokeberry juice (approx. 57.0). The color of the dried carrot depended to a greatest extent on the type of juice used for initial enrichment. The darkest chokeberry juice significantly darkened the carrot color, lowering the a* and b* parameter values, regardless of the drying method, which was visible visually and in the photos (Table S2). Excluding the samples enriched in chokeberry juice, the lightness of the L* color was less diverse than the redness a* and yellowness of the dried carrot color. Studies conducted by Yusuf et al. [22] on convection-microwave-vacuum drying (CD-MVD) of four colored carrot varieties (white, purple, yellow, and orange) preceded by osmotic dehydration showed that samples dehydrated in chokeberry concentrate were characterized by significantly lower color lightness L* compared to samples dehydrated in apple or cherry concentrate.

This is due to the high content of anthocyanins in chokeberries, which increases the darkness of the product. Most of the freeze-dried samples, including the control one, showed comparable or higher lightness of the color than the color of fresh carrots. In terms of the tested color parameters a* and b*, the convectively dried samples (27.7–32.8 and 21.9–38.4, respectively) and freeze-dried samples (18.6–29.0 and 26.1–58.1, respectively), excluding enrichment in chokeberry juice, were characterized by smaller color changes, and the values of some indicators were even higher than the color of the raw material.

The color parameters of the samples dried using the microwave-vacuum method in different variants were characterized by a much greater diversity; in terms of color lightness from 28.8 to 58.0, in the range of the a* parameter from 3.6 to 26.8 and the b* parameter from 4.5 to 50.2 (Tables S1 and S2).

In most samples, lower color lightness was observed in samples pre-enriched in chokeberry and pomegranate juice and higher in carrots dried after enrichment in sea buckthorn juice and after blanching, convective drying, and without pre-treatment. Some samples enriched in sea buckthorn juice and blanched were characterized by higher redness. In most samples dried by the microwave-vacuum method, the redness parameter was, however, significantly lower than the raw material color, not exceeding the value of 20, including many samples with a value below 10. In the case of the b* parameter, enrichment in sea buckthorn juice is worth noting, especially at lower microwave power and higher pressure (250 W/6.5 kPa) and higher microwave power (400 W), as well as pre-blanching. In these conditions, the yellowness of the color was significantly higher, even by about 36%, than the color of fresh carrot samples. Regardless of the drying method and MVD drying conditions, the use of chokeberry juice resulted in a significant increase in the color tone (66.8–78.5), even two-fold concerning the raw material color (35.5) (Figure 1). In most other cases, a decrease in the color tone of dried carrot was observed, even two-fold. On the other hand, color saturation values were the lowest in the absence of pre-treatment in carrots dried by the MVD method (7.6–26.4) and using pre-enrichment of carrots in chokeberry juice (13.7–26.3), as well as in carrots dried by MVD after pre-enrichment in PG juice (19.2–28.8). SB juice allowed the maintenance or enhancement of the color saturation of dried carrots. Hence, its use is particularly useful for producing dried carrot snacks. 

Color parameters are an important attribute reflecting the sensory properties and quality of dried vegetables, including snack products. Lightening the color of dried carrots could have reduced the product’s attractiveness (Table S1). The yellow–orange color of this carrot variety is also desirable after drying. The dominant factor influencing the absolute difference in the color of dried carrots was pre-enrichment in chokeberry juice, which is very dark. The use of other juices resulted in smaller color changes.

In the dried carrot samples, sea buckthorn juice and pre-blanching proved particularly useful, as they increased the redness and, in some samples, the yellowness. In general, it can be suggested that clear color changes may apply to both freeze-dried and microwave-vacuum-dried samples. The reasons for these changes are different; in the first method, the increase in color brightness dominated, and in the second, including pre-treatment, changes in redness and yellowness. Increasing yellowness by enriching with sea buckthorn juice or blanching carrots proved particularly beneficial. The presence of α and β-carotene is responsible for orange, red, and yellow colors. In addition, β-carotene as a precursor of vitamin A prevents diseases caused by oxidative stress [34]. As shown by Sani et al. [35], almost all available processing methods cause significant degradation of carotenoids. Therefore, it is important to preserve carotenoids by pre-blanching carrots or enriching them in juices, which are a good source of natural ingredients and thus improve the color and health-promoting properties of dried carrots. 

### 2.2. Influence of the Osmotic Pre-Treatment, Drying Method, and Microwave-Vacuum-Drying Parameters on Chemical Properties of Dried Carrot

#### 2.2.1. Carotenoid Content of Fresh and Dried Carrot

The presence of pigments, mainly carotenoids, lycopene, and anthocyanins, is based on the color of carrot varieties [36]. Orange and dark orange carrots are rich in lutein, α-carotene, and β-carotene, red carrots are rich in lycopene, and purple carrots are rich in anthocyanins and phenolic compounds [37]. As shown by Saini et al. [35] and other researchers [38,39], carotenoids and their derivatives play an important role in plants, animals, and people, from being a cellular antioxidant to gene regulation, i.e., at the cellular and molecular level.

The total content of carotenoids in fresh carrots was 37.2 mg/100 g d.m., including 10.3 mg/100 g d.m. of α-carotene and 25.9 mg/100 g d.m. of β-carotene (Table 3). This value is within the range given by Ahmad et al. [40], i.e., 3.2–170 mg/kg. Due to both color and nutritional value, carotenoid content is crucial in determining the thermal quality of dried carrots [41]. Simultaneously, they are sensitive to heat, oxygen, light, and enzymes. A significant effect of the drying method and the pre-treatment on the content of individual carotenoids and their total amount in dried carrots was found. Because the same factors significantly influenced the content of individual carotenoids and their sum in dried samples, the discussion of results focused mainly on the total content of carotenoid pigments. The content of carotenoid pigments in the dried products obtained was varied. It ranged from 2.3 mg/100 g d.m. for microwave-vacuum-dried product (6.5 kPa/400 W) initially enriched with pomegranate juice components to 35.4 mg/100 g d.m. for convective-dried product subjected to microwave pre-blanching. Both drying and pre-treatment resulted in a decrease in the total content of carotenoids in dried carrot products compared to the raw material before drying. Convective-dried products were characterized by a significantly higher content of carotenoid pigments (14.2–35.4 mg/100 g d.m.). The lowest and highest content of carotenoids within this drying method were recorded, respectively, for the convective-dried product initially enriched with pomegranate juice components and for the carrot sample subjected to microwave pre-blanching.

However, no statistically significant difference was found between the total carotenoid content in convectively dried carrots and the raw material. Research conducted by Ramesh et al. [42] showed that microwave blanching reduced the loss of total carotenoid content by up to 35.7% compared to carrots blanched in water. A significantly lower average content of carotenoid pigments was characteristic of freeze-dried samples (8.1 mg/100 g d.m.) and microwave-vacuum-dried samples (14.1 mg/100 g d.m.). Considering the pre-treatment before drying, a significantly lower average content of carotenoids was noted for samples pre-enriched with pomegranate juice components (9.2 mg/100 g d.m.) and chokeberry (10.2 mg/100 g d.m.) and a higher one for samples enriched with sea buckthorn juice (17.4 mg/100 g d.m.). Enrichment in sea buckthorn juice allowed for a 41.4% and 46.8% increase in the average content of carotenoids compared to pomegranate and chokeberry juice. A significantly higher average level of carotenoid pigments was found in the samples subjected to pre-blanching in water (21.5 mg/100 g d.m.) and a slightly lower level after pre-blanching in the microwave (18.5 mg/100 g d.m.). No significant effect of pressure and microwave power of microwave-vacuum drying on the value of the discussed parameter in carrot samples was demonstrated. However, certain trends were observed. The highest and lowest average content of carotenoid pigments was noted in dried carrots obtained at pressures of 3.5 and 6.5 kPa and at a microwave power of 400 W. The average content of carotenoids in these conditions was 15.3 mg/100 g d.m. (3.5 kPa/400 W) and 13.5 mg/100 g d.m. (6.5 kPa/400 W). However, when a lower microwave power of 250 W was used, the average total content of carotenoids at 3.5 and 6.5 kPa was similar, 13.6–13.9 mg/100 g d.m. In the study by Mendonça et al. [43], two microwave power levels were used for drying a 3 mm thick sheet of carrot paste measuring 150 mm × 100 mm using the MVD method, either at a constant level or in two stages. It was shown that the highest β-carotene retention was observed, in the case of the single stage, the lowest microwave power level used (87.4%), and the highest microwave power level in the two stage (84.3%).

The retention of carotenoids depended to the greatest extent on the drying and blanching method, as well as osmotic enrichment in SB juice. Other researchers confirmed the influence of these factors. In the study by Liu et al. [44], pre-blanching with hot water, steam, or microwave affects the reduction of enzyme activity, including polyphenoloxidase (PPO) and peroxidase (POD) in raw plant materials. This can reduce the browning of dried samples [45]. When drying carrot products, thermostable enzymes that may be responsible for carotene degradation should also be considered. Based on research on the drying of various plant raw materials during the drying process, carotenoids are degraded due to the action of temperature, oxygen, and light, which was also confirmed by da Silva Moura et al. [46] in their studies on convective drying of Jambu leaves (*Acmella oleracea*), some kind of vegetable used in human food. In the studies of Prieciņa and Kārkliņa [47], it was shown that drying carrots contributed to an increase in the content of carotenoids compared to fresh carrots (190.7 mg β-carotene/100 g d.m.). Higher carotenoid content was obtained for microwave-vacuum-dried samples (282.9 mg β-carotene/100 g d.m.) compared to convective drying (200.5 mg β-carotene/100 g d.m.). 

Convective drying without pre-treatment at 60 °C, as well as after microwave blanching and impregnation with SB juice, allowed for obtaining the highest values (28.4–34.4 mg/100 g d.m.), lower by 4.6–23.7% than the content of carotenoids in the raw material. Application of selected MVD drying parameters (MVD_6.5_400_M_BL) resulted in a reduction of carotenoid content by about 18.2%. Freeze-drying, considered as a reference in terms of the quality of dried samples, did not have a positive effect on the preservation of carotenoids in dried carrots. Each treatment, especially microwave blanching (4.9–15.9 mg/100 g d.m.) before FD, resulted in greater preservation of carotenoids (13.2–42.79%) compared to drying without treatment (3.2 mg/100 g d.m., 8.6% preservation). In most (approximately 67%) of the samples dried using the MVD method, the carotenoid content was greater than 10 mg/100 g d.m., which indicated a much greater (27.5%) preservation of carotenoids compared to the FD method. As mentioned earlier, different processing methods cause carotenoid degradation [35]. To preserve them, it is necessary to blanch carrots or enrich them in juices, which are a good source of natural ingredients, and in this way also improve the color and health-promoting properties of dried carrots. In the case of carrot drying, the use of FD is less justified than MVD. However, due to the uneven effect of microwaves on the dried material, selecting drying conditions using the MVD method is not easy and requires refinement [32]. Another study [48] conducted to investigate the effect of hot-air drying and freeze-drying of carrots pretreated with 4% potassium metabisulphite on the content of bioactive components showed that sulphite as an antioxidant present in KMS stabilizes carotenoids, and their increase was found in the case of hot-air drying by about 30% compared to the untreated sample. However, no significant difference was observed in the case of freeze-dried samples. 

#### 2.2.2. Total Phenolic Content (TPC) of Fresh and Dried Carrot

**Figure 3:**
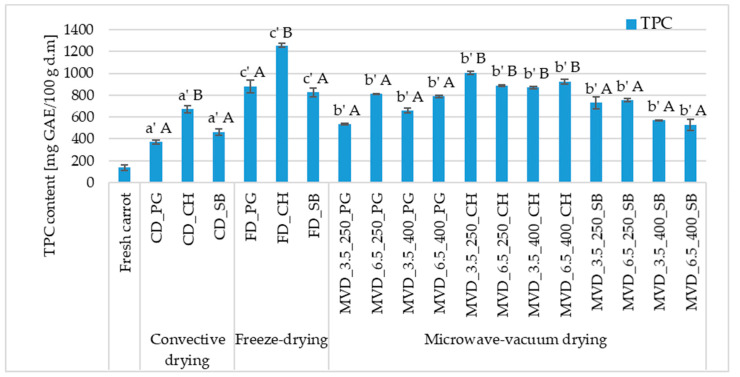
The effect of the type of osmotic pre-treatment (NFC juice), drying method, and microwave-vacuum-drying parameters on the total phenolic content (TPC) of dried carrot samples. Different letters a’, b’, c’ (type of drying method); A, B (type of NFC juice)—homogeneous groups show the statistical difference at *p* ≤ 0.05. When all the data were in one homogenous group (*p* > 0.05), a’, A letters were omitted. Code designations of dried samples are as in the caption of Table 1.

The total phenolic content of dried carrot samples increased significantly (even by 89.0%) compared to the raw material (138.0 mg GAE/100 g d.m.) (Figure 3). Carrots are a good source of phenolic compounds, highly concentrated in the periderm tissue. In carrot skin, which constitutes 11% of the fresh weight, their content reaches 54.1%, while in phloem tissue, it is 39.5% and xylem 6.4%. Orange carrots have fewer phenolic compounds than purple carrots, which can reach 33.25 mg/g d.m. [37,49]. During thermal processes, food components such as proteins, amino acids, and sugars are the main substrates of the Maillard reaction. Polyphenols that also occur in food participate in the regulation of this reaction, being substrates of its initial, intermediate, or final stages. The increase in TPC content in dried carrots may be caused by the formation of Maillard reaction products, which lead to the formation of new phenolic compounds. In addition, studies indicate that phenolic compounds with higher molecular weight can be released into simpler forms by thermal treatment, such as drying, which leads to an increase in the total phenolic content in such samples [50,51]. 

The values of this indicator in dried carrots depended significantly on the drying method and the type of pre-treatment. Statistical analysis did not show a significant effect of microwave-vacuum-drying conditions on the value of the parameter discussed (Figure 3). The highest mean TPC was characteristic of carrot lyophilizes (986.9 mg GAE/100 g d.m.), a slightly lower value was noted for microwave-vacuum-dried samples (755.0 mg GAE/100 g d.m.), and the lowest after convective drying (501.0 mg GAE/100 g d.m.). Studies by Prieciņa and Kārkliņa [47] on microwave-vacuum drying of carrots after initial steam blanching (1.5 and 3 min) and convection, as a comparative method, also showed an increase in the total polyphenol content. In fresh carrots, the result was recorded at the level of 114.4 mg GAE/100 g d.m, and for samples steam blanched for 1.5 and 3 min, respectively, 403.0 and 385.7 mg GAE/100 g d.m. The most significant increase in the total polyphenol content (TPC) was observed in convection-dried samples (593.1 mg GAE/100 g d.m.). The dominant effect was using osmotic enrichment in chokeberry juice, which caused a significant increase in TPC, regardless of the drying method. Among the samples enriched with CH juice, the highest values were found in freeze-dried carrots (1255.0 mg GAE/100 g d.m.), 25–44% lower in microwave-vacuum-dried products and about 2 times lower after convection drying.

A separate homogeneous group (A) was formed by dried products obtained from enrichment with SB and PG juice, where the average value of the discussed indicator was 644.8 and 674.0 mg GAE/100 g d.m., respectively. Although the statistical analysis did not show a significant effect of microwave-vacuum-drying parameters, i.e., microwave pressure and power, on the TPC value in dried carrot products, trends were observed. The dried material obtained at a pressure of 3.5 kPa and microwave power of 400 W had a lower average TPC content, and the dried material obtained at a pressure of 6.5 kPa and microwave power of 250 W had a higher average TPC content. The average TPC value in these conditions was 699.0 mg GAE/100 g d.m. (3.5 kPa/400 W) and 817.0 mg GAE/100 g d.m. (6.5 kPa/250 W). Lower microwave power and higher pressure caused an increase in the TPC content in the dried carrot. 

Regarding TPC content in dried carrots, both pre-treatments and their absence before each type of drying were beneficial because drying caused the concentration of these compounds in the dried carrots. Compared to the raw material, the TPC content in the dried carrots increased by 3–9 times. Drying by the FD and MVD methods gave a similar effect of increasing the TPC content, including a greater effect after enrichment in CH juice. Various factors could influence the increase in the TPC content in the dried carrots.

#### 2.2.3. Anthocyanins Content of Fresh and Dried Carrot

The anthocyanins profile was assessed in the raw material and selected dried carrot samples (Table 4). Fresh carrots and those dried using various methods did not contain these compounds, but the application of pre-enrichment in chokeberry NFC juice influenced the obtaining of these compounds in the tested dried carrots. The content of components such as cyanidin-3-O-galactoside (Cy-3-O-gal), cyanidin-3-O-glucoside (Cy-3-O-glu), cyanidin-3-O-arabinoside (Cy-3-O-ara), and cyanidin-3-O-xyloside (Cy-3-O-xyl), expressed in mg/100 g d.m., was determined in carrot samples dried using various techniques after pre-osmotic enrichment in NFC juices. The presence of these compounds was not detected in the raw material and samples enriched in pomegranate and sea buckthorn juice; their values were below 0.01 mg/kg. The effect of the drying method on their content in carrots was found. The highest content of these compounds was shown in samples dried by freeze-drying. In these samples, total anthocyanins amounted to about 22.6 mg/100 g d.m. The first three compounds were present in dried samples obtained by the microwave-vacuum method in small amounts or marginally, and only Cy-3-O-xyl was in the range of 2.0–2.8 mg/100 g d.m., while in the remaining samples dried by freeze-drying and convection, it was about 4.1 and 2.7 mg/100 g d.m., respectively.

#### 2.2.4. DPPH• and ABTS•+ Antioxidant Activity of Fresh and Dried Carrot

**Figure 4:**
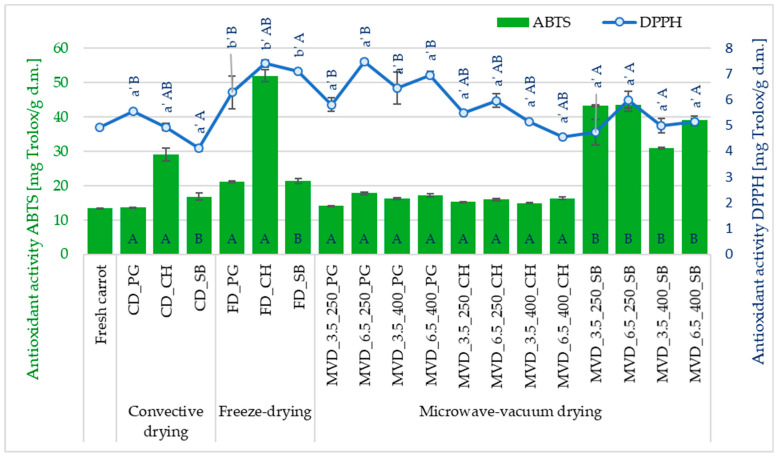
The effect of the type of osmotic pre-treatment (NFC juice), drying method, and microwave-vacuum-drying parameters on the antioxidant activity (DPPH• and ABTS•+). Different letters a’, b’ (type of drying method); A, B (type of NFC juice)—homogeneous groups show the statistical difference at *p* ≤ 0.05. When all the data were in one homogenous group (*p* > 0.05), a’, A letters were omitted. Coding markings of dried samples are as in Table 1.

The values of total antioxidant capacity measured against the ABTS radical were higher than those measured against the DPPH• radical (Figure 4). According to the ABTS method, the antioxidant activity ranged from 13.3 to 52.0 mg Trolox/g d.m., and DPPH• from 4.1 to 7.5 mg Trolox/g d.m. According to Minutti-López Sierra et al. [52], this is probably because the ABTS radical measures the absorbance of both lipophilic and hydrophilic compounds, while the DPPH• radical only of hydrophobic compounds.

The type of drying method used significantly influenced (*p* < 0.05) the antioxidant capacity of dried carrots towards the DPPH• radical. Also, in the study by Tabtiang et al. [53], it was found that microwave-assisted drying is more beneficial compared to convective drying and even vacuum freeze-drying. Statistical analysis showed a significant effect of the type of NFC juice used (*p* < 0.05) and no effect of microwave-vacuum-drying conditions (*p* > 0.05) on the antioxidant activity of carrots measured towards the DPPH• and ABTS•+ radicals. The highest average antioxidant activity towards the DPPH• radical was noted for freeze-dried samples (6.9 mg Trolox/g d.m.). Slightly lower values at 4.9 and 5.7 mg Trolox/g d.m. were shown by convectively and microwave-vacuum-dried carrot samples, respectively.

Osmotic enrichment of carrots with PG juice components (6.4 mg Trolox/g d.m.) contributed to obtaining significantly higher average antioxidant activity of DPPH• dried carrots compared to carrots enriched with SB juice (5.3 mg Trolox/g d.m.). The antioxidant activity DPPH• of dried carrots enriched with CH juice did not differ significantly and averaged 5.6 mg Trolox/g d.m. Despite the rather diversified antioxidant activity ABTS•+ of dried carrots, statistical analysis did not show a significant effect of the drying method on the value of this indicator. The average antioxidant activity of ABTS•+ in the tested dried products in the setting from the lowest to the highest value, depending on the type of drying method, was as follows: freeze-drying (FD; 30.0 mg Trolox/g d.m.) ⇒ microwave-vacuum method (MVD; 23.7 mg Trolox/g d.m.) ⇒ convection (CD; 18.6 mg Trolox/g d.m.). The values of the average antioxidant activity of ABTS•+ dried carrot products enriched with PG and CH juice did not differ significantly; they were 15.9 and 16.2 mg Trolox/g d.m., respectively. A separate homogeneous group (B) was formed by dried products obtained from enrichment with SB juice with an average value of 39.6 mg Trolox/g d.m.

Although statistical analysis did not show a significant effect of microwave-vacuum-drying parameters on the antioxidant activity of DPPH• and ABTS•+, certain trends were observed. The lowest and highest average antioxidant activity of DPPH• was noted in dried carrots obtained at a pressure of 3.5 and 6.5 kPa and at a microwave power of 250 W. The average antioxidant activity of DPPH• under these conditions was 5.3 mg Trolox/g d.m. (3.5 kPa/250 W) and 6.5 mg Trolox/g d.m. (6.5 kPa/ 250 W). However, when a higher microwave power of 400 W was used, the average antioxidant activity of DPPH• at a pressure of 3.5 and 6.5 kPa was 5.5 mg Trolox/g d.m. In the case of ABTS•+ activity, the lowest average antioxidant activity was noted in dried carrots obtained at a pressure of 3.5 kPa and microwave power of 400 W, and the highest at a pressure of 6.5 kPa and a microwave power of 250 W. The average antioxidant activity of ABTS•+ under these conditions was 20.7 mg Trolox/g d.m. (3.5 kPa/400 W) and 25.7 mg Trolox/g d.m. (6.5 kPa/ 250 W). Similarly to the TPC content of dried carrots, higher pressure and lower microwave power resulted in higher antioxidant capacity of the dried carrots measured against both radicals.

#### 2.2.5. Vitamin C Profile of Fresh and Dried Carrot

The total content of vitamin C in raw carrots and dried carrots enriched with NFC juices from pomegranate, chokeberry, and sea buckthorn was expressed as the sum of L-ascorbic acid (AA) and L-dehydroascorbic acid (DHAA). The level of vitamin C was varied and ranged from 0.99 to 44.97 mg/100 g d.m. (Table 5). Statistical analysis showed a significant effect of the type of drying method on the total content of vitamin C in the tested dried products. In terms of drying method, the highest average content of vitamin C was noted in convection-dried carrot samples (17.6 mg/100 g d.m.), significantly lower in microwave-vacuum-dried products (14.3 mg/100 g d.m.), and the lowest in lyophilized carrot (8.2 mg/100 g d.m.). Research by Turkiewicz et al. [21] on drying Japanese quince fruit using different methods also showed that microwave-vacuum-dried fruit had a higher L-ascorbic acid content than freeze-dried fruit. This may be due to the different moisture content in this dried fruit, which, in addition to oxygen and temperature, also affects the degradation of vitamin C content or the formation of new non-specific compounds under the influence of microwaves. In addition, the lowest content of ascorbic acid was recorded in the case of convective-dried fruit [21]. No significant effect of microwave-vacuum-drying parameters, i.e., microwave pressure and power, on the total vitamin C content in the tested dried products was demonstrated. However, it was noted that the use of higher pressure (6.5 kPa) contributed to an increase in the total content of vitamin C, and the highest total content of vitamin C, almost 45 mg/100 g d.m., was obtained at a pressure of 6.5 kPa and a microwave power of 400 W. It was shown that the average contents of vitamin C in dried carrots differed significantly depending on the type of NFC juice used. The use of sea buckthorn juice for the initial enrichment treatment contributed to a significant increase in the total content of vitamin C, even by 89.1%, compared to raw carrots.

#### 2.2.6. Sugar Content Profile of Fresh and Dried Carrot

**Figure 5:**
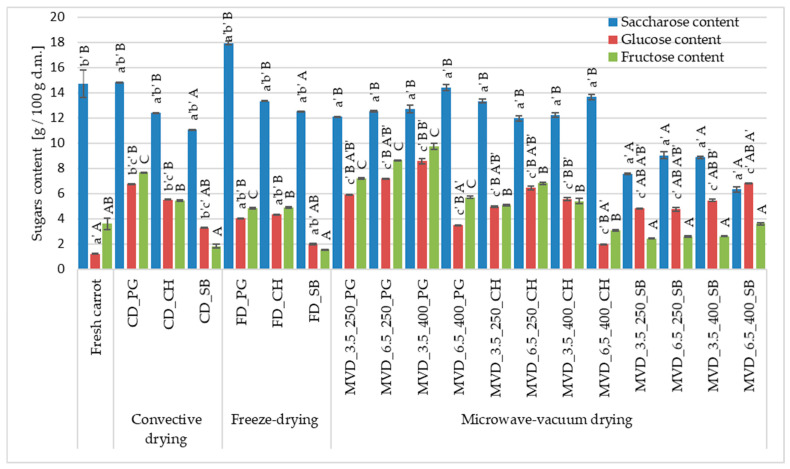
The effect of the type of osmotic pre-treatment (NFC juice), drying method, and microwave-vacuum-drying parameters on the sugar content of dried carrot samples. Different letters a’, b’, c’ (type of drying method); A, B, C (type of NFC juice) and A’, B’ (pressure and microwave power)—homogeneous groups show the statistical difference (*p* < 0.05). When all the data were in only one homogenous group, a’, A, or A’ letters were omitted. Code designations of dried samples are as in the caption of Table 1.

The total sugar content in the raw material was about 19.53 g/100 g d.m., including a saccharose content of about 75.3% and a fructose content of 18.4% (Figure 5). The influence of the MVD drying method and conditions and osmotic pre-treatment was demonstrated and also caused significant changes in the total sugar content, including the content of measured sugars, i.e., saccharose, glucose, and fructose (Figure 5). Compared to the raw material, the changes in the sugar content profile in osmotically enriched carrot consisted of a decrease in the saccharose content and an increase in the glucose and fructose content. Regardless of the drying method, in the case of osmotic pre-enrichment in pomegranate juice, the sugar content increased the most, by 49.9 and 37.4% in samples dried by convection and freeze-drying, and in the case of puffing drying by 45.5 and 59.1% in samples dried under a pressure and at a microwave power of 6.5/250 and 3.5 kPa/400 W, respectively. The obtained values depended on the drying parameters. It was observed that higher pressure (6.5 kPa) at a lower microwave power (250 W) caused an increase in the total sugar content, regardless of the type of NFC juice. At a higher microwave power, the relationship was reversed, especially in the case of using PG juice with a high glucose and fructose content (60.6–68.0 g/L), as well as saccharose (approx. 4.5 g/L).

It can be assumed that after greater penetration of these sugars, higher pressure and lower microwave power caused a greater increase in dry matter content. The use of sea buckthorn (SB) juice with a sugar content comparable to the raw material but the lowest pH caused a significant decrease in the total sugar content to approximately 24.1%. The lower pH could decompose saccharose into monosaccharides and wash them out of the carrot tissue. Compared to the raw material, the changes in the sugar content profile in osmotically enriched carrots consisted of reducing the saccharose content and increasing the glucose and fructose content.

### 2.3. Comprehensive Summary of Results

Principal component analysis (PCA) was performed on the data, separating two groups. The first group concerned the technological properties of dried carrots concerning thermal pre-treatment (blanching and convective drying), drying method, and MVD drying conditions and the second group concerned the data related to the pre-enrichment of carrots (Figure S1). Cattell’s, which shows the eigenvalues, scree plots were prepared to determine the number of principal components. In both Figure S1a,b graphs, the first components captured most of the information (steep part of the curve); therefore, the remaining ones (flattened part of the curve) were ignored as “random noise”. Moreover, in the first group of data for the first eight and in the second group for nine factors of eigenvalues ≥ 1 (Figure S1), the cumulative values accounted for 85–89% of the variance. Horizontally arranged to decrease eigenvalues indicating subsequent components extracting only “random noise” resulted in the loss of information in the variable analysis at 11–15%.

**Figure 6:**
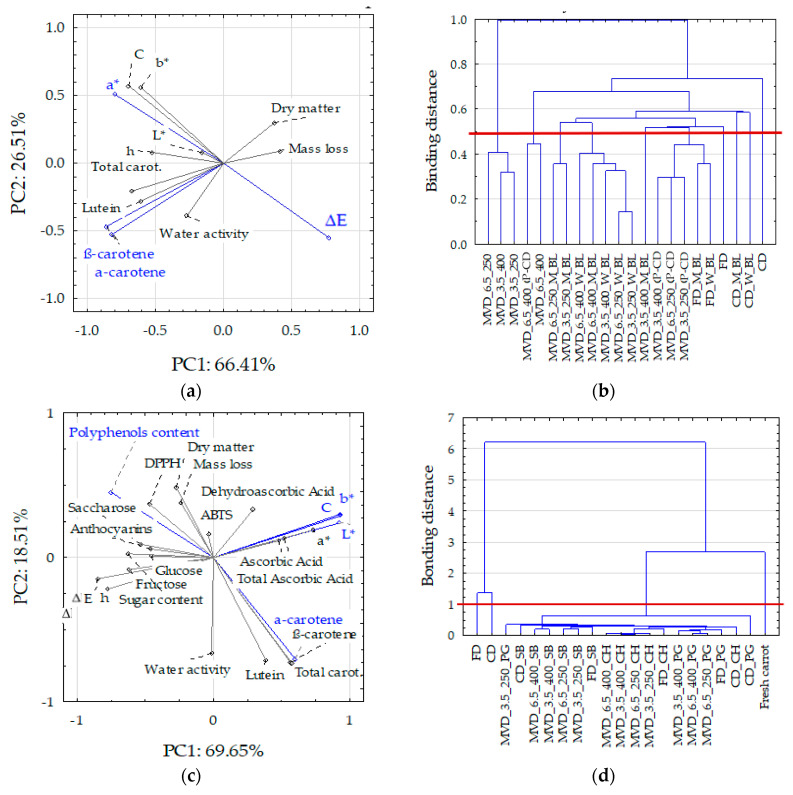
PCA and cluster analysis of technological properties related to thermal pre-treatment (blanching and convective drying) and drying method with MVD drying parameters (**a**,**b**) and data related to carrot pre-enrichment (**c**,**d**): (**a**,**c**) PCA graphs; (**b**,**d**) cluster analysis. Markings: L*, a*, b*, C, h, and ΔE—lightness, redness, yellowness, saturation, tone and absolute color difference, respectively. Blue lines (**a**,**c**) indicate active data included in the PCA analysis. Red lines separate values smaller than distances 0.5 (**b**) and 1.0 (**d**), allowing distinguishing groups of clusters with similar properties for standardized values.

In analyzing the second group of data related to the initial enrichment of carrots, the PC1 and PC2 components explained 88.2% of the variability of the dried carrot properties (Figure 6c,d). The main share of the PC1 component was taken by the color parameters, TPC, and carotenoids, except for lutein, and PC2 by carotenoids and water activity.

In the first data group (technological properties), the principal components (PC1 and PC2) explained 92.2% of the variability of the properties of dried carrots. PC1 consisted of variables such as carotenoid content indices, mainly α and β carotene, as well as the color parameter a* (redness) and the absolute color difference ΔE (Figure 6a). Based on the distribution of variables in the PCA system, it resulted that the carotenoid content correlated positively with the water activity of dried carrots and inversely proportionally with the loss of mass and dry matter content. This means that decreased water activity was associated with decreased carotenoid content. On the other hand, the carotenoid content did not depend significantly on the changes in the color parameters. It confirms the results presented above (Table 1, Figure 2). The cluster method was used to compare the dried carrot samples, depending on the pre-treatment and drying method (Figure 6b). For standardized values below the connecting distance of 0.5, six cluster groups with similar properties were determined. The first three groups included carrot samples dried by convection, successively, without treatment, then blanched in water and microwave. The fourth group included three samples dried in a microwave-vacuum without pre-treatment at a microwave power of 250 and 400 W under a pressure of 6.5 kPa and at a power of 400 W under a pressure of 3.5 kPa. The fifth group of clusters consisted of two samples dried by the MVD method at a microwave power of 400 W under 6.5 kPa, including pre-convection drying. The sixth group included the remaining samples, including carrots dried by freeze-drying and by the MVD method after pre-blanching and convection drying. The greatest differences occurred between samples dried by the convection method (the first three groups of clusters) and dried by the MVD method without pre-treatment. These samples differed significantly in terms of color parameters. The absolute difference in color between convection-dried and blanched samples was lower, and these samples had the highest carotenoid contents (21.7–35.4 mg/100 g d.m.), 5–50% lower compared to the content in the raw material. Among the remaining samples, only some MVD-dried samples had carotenoid contents higher than 20 mg/100 g d.m.

Carotenoids correlated negatively with the content of total phenolic and dry matter content and mass loss and positively with water activity. Similarly to the first series of studies (Figure 6a,b), the greater drying effect associated with the increase in dry mass content and mass loss was associated with the loss of carotenoids compared to their content in the raw material. However, no correlation was found between the content of carotenoids and the changes in the color of dried carrots. This resulted from the large variation in the content of these components and the uniformity of the color of the samples (Table 2, Figure S1), especially caused by the action of microwaves in MVD drying. The content of phenolics (TPC) was negatively correlated with the content of carotenoids (r = −0.47–0.60), which means that their higher content in dried carrots was associated with a lower content of carotenoids. Their higher content caused a decrease in yellowness and color saturation (negative correlation with b* and C) but an increase in the tone and absolute color difference (positive correlation with h and ΔE), as well as antioxidant activity determined by the DPPH• method (r = 0.76) and also the anthocyanins content (r = 0.51). Moreover, DPPH• activity was positively correlated with the content of sugars and negatively with the content of lutein. On the other hand, ABTS•+ activity was positively correlated with the content of vitamin C and anthocyanins. This is understandable because both indicators of antioxidant activity allow for the detection of the antioxidant activity of other compounds [40]. Cluster analysis (Figure 6d) showed four groups with similar properties at a linking distance of 1. The first group consisted of the properties of fresh carrots, the second and third of convection-dried and freeze-dried carrots, and the last group consisted of the remaining samples dried by the MVD method, among which can be divided into those enriched in individual juices. Such similarities and differences lead to the conclusion that FD drying can be successfully replaced by the MVD method. 

## 3. Materials and Methods

### 3.1. Material and Experimental Procedure

**Figure 7:**
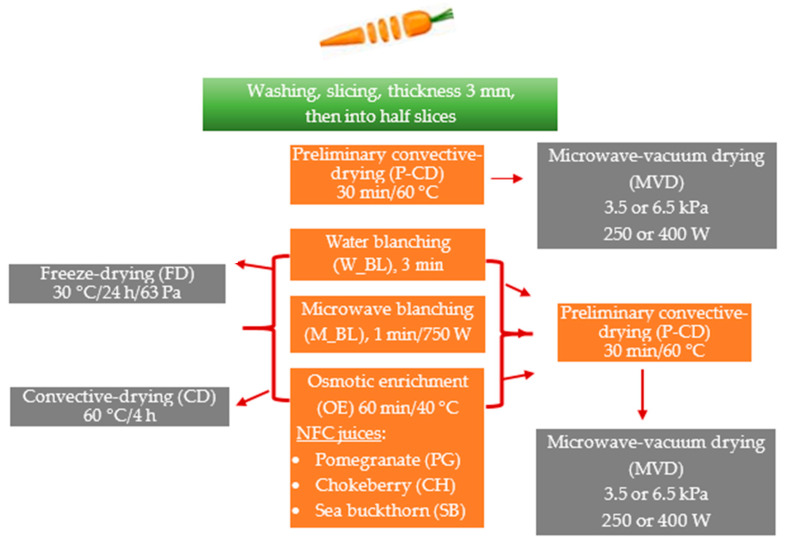
Scheme of the experimental procedure for drying carrots, including pre-treatment; the abbreviations used to code the samples are in brackets.

The research material was carrots from the “Tadeusz Karaś” horticultural farm purchased in a large-format store in Warsaw. Until the tests were carried out, the raw materials were stored at a refrigerated temperature of approximately 4 °C and a relative air humidity of 85–90%. Carrots (C) with a diameter of about 2 cm were washed and then, without removing the skin, cut into 3 mm thick slices using a slicer (Robot Coupe CL50, Vincennes, France) and then manually into half-slices. The samples were subjected to preliminary convective drying (P-CD), water blanching (W_BL), microwave blanching (M_BL), and osmotic enrichment in NFC juices from pomegranate (PG), chokeberry (CH), and sea buckthorn (SB) (Table 6 and Table 7). Then, the carrot samples were dried using three drying methods (Figure 7), i.e., microwave-vacuum drying (main method), convection drying, and freeze-drying (comparative methods).

The coefficients such as dry matter content (%), water activity (–), mass loss after drying (%), color parameters, and carotenoid profile were tested in all samples, including fresh and control samples (without pre-treatment). Additionally, mass loss was tested after pre-treatment (%). To demonstrate the effect of osmotic enrichment of carrots in NFC juices, the total phenolic content TPC, antioxidant activity (DPPH• and ABTS•+), and ascorbic acids were measured in these enriched samples and dried using different methods, with reference to the fresh sample. The content of selected anthocyanins was measured only in samples enriched with chokeberry juice because the remaining samples did not show their content or were borderline. Sugars (glucose, fructose, saccharose) were measured in all enriched samples, which involved the assessment of the effect of juice with different pH on the changes in the content of these sugars.

### 3.2. Technological Methods

#### 3.2.1. Pre-Treatment Methods

Water blanching of carrots was carried out in boiling water by immersing approximately 100 g of carrots for 3 min. After blanching, the carrot half-slices were cooled by momentary immersion in cold water. Then, the carrots were dried on filter paper. Microwave blanching of carrots was carried out for 1 min in a microwave oven (G2711N, SAMSUNG, Seoul, South Korea) at a microwave power of 750 W. Then, the samples in closed dishes were cooled for 2 minutes in a refrigerator at a temperature of approximately 5 °C. Blanched samples were weighed on a technical scale (Type WPE 2000, RADWAG, Radom, Poland) with an accuracy of 0.1 g.

The osmotic enrichment process was carried out in a water bath with a shaker (JW ELECTRONIC type T-OSM, Warsaw, Poland) maintaining the set temperature. Three types of not from concentrate (NFC) juices (producer Premium Rosa, Złotokłos, Poland) were used for enrichment: pomegranate (PG), chokeberry (CH), and sea buckthorn (SB) (Table 6 and Table 7). The ratio of sample mass to juice mass was 1:2. Osmotic enrichment was carried out at 40 °C for 60 min. After the enrichment process, the carrot half-slices were separated from the juice solution using a sieve and then dried on filter paper. Samples prepared in this way were weighed on a technical scale (Type WPE 2000, RADWAG, Radom, Poland) with an accuracy of 0.1 g.

Convective drying (CD) for pre-treatment of selected samples was carried out at a co-current airflow of 2 ± 0.1 m/s and a temperature of 60 ± 3 °C for 30 min in a laboratory convection dryer available at the Institute of Food Sciences of the Warsaw University of Life Sciences.

#### 3.2.2. Drying Methods 

The microwave-vacuum-drying (MVD) process was carried out using a dryer from PROMIS-TECH (Wrocław, Poland). In the beginning, attempts were made to dry carrots using four drying cycles, a reduced pressure of 3.5 or 6.5 kPa, a microwave power of 250 or 400 W, and a temperature of 70 °C to select the drying time depending on the quality of the obtained dried product in terms of degree of drying and color (Table 8). Using a type K thermocouple (NiCr-Ni) at the outlet from the drying chamber, the temperature of the steam removed from the dried sample was measured. When the steam temperature rose above 70 °C, the microwave oven was automatically turned off.

Convective drying (CD) was carried out with a co-current airflow of 2 ± 0.1 m/s and a temperature of 60 ± 3 °C for 4 h in a laboratory convection dryer available at the Institute of Food Sciences of the Warsaw University of Life Sciences.

Freeze drying (FD) of fresh and pre-treated carrot half-slices was carried out after freezing the samples in a shock freezer (Shock Freezer HCM 51.20, Irinox, Treviso, Italy) with airflow at −40 °C for 4 h. The frozen samples were transferred directly to the freeze dryer and dried in an Alpha 1–4 LSC freeze dryer from Christ (Osterode am Harz, Germany) for 24 h at a heating shelf temperature of 30 °C, a pressure inside the chamber of 63 Pa, and a safety pressure of 103 Pa.

### 3.3. Physical Determination

#### 3.3.1. Dry Matter Content and Water Activity

The dry matter content was determined in fresh carrots and after pre-treatment and drying using the drying method in a laboratory dryer (SUP-65 WG, WAMED, Warsaw, Poland) at a temperature of 105 °C until constant mass for about 3 h following AOAC 920.15, 2002. The measurement was performed twice.

Water activity was measured using an AQUALAB CX-2 device (Decagon Devices Inc., Pullman, WA, USA) at 25 ± 1 °C. The measurement was performed twice [25].

#### 3.3.2. Color Parameters

The color was measured using the reflectance method using a CR-5 colorimeter (Konica Minolta Bench-top, Japan) in the CIELab system. Concerning the color parameters of raw carrots as a standard, the absolute color difference ΔE was calculated from Equation (1).

(1) $$\Delta E=\sqrt{({L-L_{0})}^{2}+({a-a_{0})}^{2}+({b-b_{0})}^{2}}$$

A standard D65 light source, a standard observer of 2°, and a measuring aperture of 3 mm were used. Before the analysis, equipment calibration was performed using a white and black reference plate. The measurement was performed in 5 repetitions. To eliminate the influence of color in different locations of the carrot, the samples were crushed into smaller particles (±3 mm) and appropriate portions were taken randomly. 

### 3.4. Chemical Determination

#### 3.4.1. Extraction to Determine Bioactive Compounds and Antioxidant Activity

Raw carrots and the analyzed sample variants were extracted to determine the content of bioactive compounds and antioxidant activity. The samples were ground in a laboratory grinder (IKA A11 basic; IKA-Werke GmbH, Staufen, Germany), and then approximately 0.5 g of the sample was weighed into falcons with an accuracy of 0.0001 g on an analytical balance (Sartorius ME 235S Genius Analytical Balance, Kostrzyn Wielkopolski, Poland). Samples were extracted by weight by adding 10 g of 80% methanol solution acidified with 0.1% HCl. Then, they were mixed and subjected to ultrasound in an ultrasonic bath (MKD-6, Ultrasonic, Poland) for 10 min at a frequency of 45 kHz and a temperature of 25 °C. Subsequently, the samples were shaken in an orbital shaker (SK-O330-PRO by DLab, Shunyi, China) for 10 min at a rotation speed of 500 rpm. The supernatant was centrifuged in a laboratory centrifuge (Rotina 380R, Hettich, Tuttlingen, Germany) for 5 min at a rotation speed of 6500 rpm and a temperature of 4 °C. The extraction was carried out three times, and the solution above the sediment was collected into a volumetric flask with a volume of 25 mL. After the extraction, the flasks were filled to the mark with the solvent solution. Two extracts were made for each sample.

#### 3.4.2. Carotenoids Content

All analyzed samples were subjected to the extraction of carotenoid compounds using the method proposed by Mapelli-Brahm et al. [54] with some modifications. Approximately 0.3 g of sample was weighed into the falcons on an analytical scale (Sartorius ME 235S Genius Analytical Balance, Kostrzyn Wielkopolski, Poland). The first extraction was performed by adding 1 mL of distilled water, 3.5 mL of hexane, 1 mL of BHT (500 mg of BHT in 1 L of hexane), and 4.5 mL of acetone to the falcons. The prepared mixture was subjected to ultrasound in an ultrasonic bath (45 kHz, 200 W, 25 °C; MKD-6 Ultrasonic, Poland) for 5 min and then centrifuged (Rotina 380R, Hettich, Tuttlingen, Germany) at 2500× *g* at a temperature of 4 °C for 5 min. The organic phase was collected and transferred to a 50 mL centrifuge falcon. Subsequent extractions of the aqueous phase were carried out 3–4 times using 4.5 mL of hexane and 2 mL of an aqueous solution saturated with NaCl. The collected organic phase was mixed, then 1.5 mL was taken into round-bottom flasks and evaporated (Rotavapor R-300, Buchi, Switzerland) at 30 °C and 160 mbar. A total of 1.5 mL of acetone was added to the evaporated samples, mixed, and filtered into vials through a 0.45 µm syringe filter (Macherey-Nagel, Düren, Germany). Samples prepared in this way were analyzed on an HPLC apparatus (Waters, Milford, MA, USA) equipped with a Waters 2695 separation module (pump, autosampler, and degasser) and a 2995 photodiode array detector. The wavelength λ = 450 nm was used to quantify α- and β-carotene and lutein. The analysis was performed using an YMC Carotenoid column (3 µm, 4.6 mm C × 150 mm) heated to 25 °C. The injection volume was 25 μL, and the mobile phase flow rate was 1.0 mL/min. A single sample was analyzed for 75 min. The mobile phase solvents used were as follows: phase A—methanol solution with 0.1% ammonium acetate and phase B—methyl tert-butyl ether, with the following gradient: from 0 to 44 min, 100% (A); 45–54 min, 85% (A) and 15% (B); 55–59 min, 40% (A) and 60% (B); 60–69 min, 30% (A) and 70% (B), and finally 70–75 min, 100% (A). The results are given in mg of β-carotene equivalents per 100 g of dry matter content of the tested sample from the calibration curve prepared for various concentrations of β-carotene. The analysis was performed in duplicate.

#### 3.4.3. Total Phenolic Content

The supernatant used to determine the content of total phenolics was prepared following Section 3.4.1. The content of phenolic compounds was determined spectrophotometrically (6705 UV–vis Spectrophotometer, Jenway, UK) using the method proposed by Gao et al. [55]. To 0.2 mL of the extract, 0.4 mL of Folin–Ciocalteu reagent, 4 mL of distilled water, and 2 mL of 15% sodium carbonate solution were added. The contents of the samples were mixed and then incubated in the dark for 60 min. After this time, the absorbance of the samples was measured at a wavelength of λ = 765 nm against a blank sample. The results were expressed in mg of gallic acid equivalents (GAE) per 100 g of dry sample (d.m.). The analysis was performed twice.

#### 3.4.4. Anthocyanins Content

The method presented by Oszmiański [56] was used to determine the anthocyanin content in the samples. Prepared following Section 3.4.1., the extracts were filtered through a syringe filter of 0.45 µm (Macherey-Nagel, Düren, Germany) into vials. The separation was performed in a liquid chromatograph (Waters, Milford, MA, USA) equipped with a Waters 2695 separation module (pump, autosampler, and degasser) and a 2995 photodiode array detector. The column used for analysis was a Sunfire C (18.5 µm, 4.6 mm × 250 mm) reversed-phase column (Waters models, Milford, MA, USA) and a Sun-fire C18 Sentry precolumn, 5 µm, 4.6 mm × 20 mm (Waters, Milford, MA, USA). The column was heated to 25 °C, and the sample temperature was maintained at 5 °C. The sample injection volume was 10 µl, and the analysis time was 26 min. Samples were eluted with 4.5% aqueous formic acid (A) and 80% acetonitrile in the previous formic acid solution (B) using a flow rate of 1.0 mL/min. The anthocyanin content was quantified at 520 nm, and the amount of anthocyanin was expressed as cyanidin 3-glucoside. The analysis was performed twice.

#### 3.4.5. Vitamin C Content

To determine the content of vitamin C in samples enriched with NFC juices and raw carrots, expressed as L-ascorbic acid (AA) and L-dehydroascorbic acid (DHAA), the method described by Odriozola-Serrano et al. [57] was used. Approximately 1.0 g of the microwave-vacuum- and convection-dried sample and approximately 0.3 g of the lyophilisate were weighed into a 10 mL flask and then supplemented with a 0.01% (*v*/*v*) phosphoric acid solution. The flasks were left to stand for 15 min; the solution was transferred to Eppendorf tubes and then centrifuged in a centrifuge for 2 min at a rotation speed of 1200 rpm. The obtained supernatant was filtered through syringe filters with a pore diameter of 0.45 μm. Next, 0.5 mL of the extract and 0.5 mL of 0.01% (*v*/*v*) phosphoric acid (AA determination) or 0.5 mL of a solution of dithiothreitol (DTT) in 0.01% phosphoric acid (determination of the sum of AA and DHAA) were added to the vials. The HPLC system and column were the same as described in Section 3.4.2. Then, 10 μL of the sample was eluted isocratically with 0.01% (*v*/*v*) m-phosphoric acid for 10 min at a flow rate of 1.0 mL/min. The column temperature was 25 °C, and the sample temperature was 5 °C.

#### 3.4.6. Antioxidant Activity Determined with DPPH•

Antioxidant activity expressed towards the DPPH• radical (2,2-diphenyl-1-picrylhydrazyl) was determined by the spectrophotometric method (6705 UV–vis Spectrophotometer, Jenway, UK) according to the methodology presented by Yen and Chen [58] with some modifications. A stock solution of the DPPH• radical at a concentration of 1 mM was prepared in an 80% (*v*/*v*) methanol solution 3 h before the analysis and incubated at room temperature without access to light. After this, the solution was diluted with 80% (*v*/*v*) methanol to obtain a concentration of 0.1 mM and absorbance in the range of 0.700–0.800, at a wavelength of λ = 517 nm. A standard curve was prepared by dissolving Trolox in methanol to obtain a concentration of 1 mg/mL. Then, standard solutions with concentrations of 10, 20, 30, 40, 50, and 100 µg/mL were prepared from the prepared solution in 10 mL volumetric flasks. Up to 0.1 mL of the extract solution was obtained following Section 3.4.1. A total of 2 mL of radical solution was added, mixed, and incubated for 30 min in a dark place at room temperature. Then, the absorbance was measured at a wavelength of λ = 517 nm. Antioxidant activity based on the ability of carrot extracts to scavenge DPPH• free radicals was expressed as µM Trolox per 100 g of dry matter (d.m.) of the sample. The analysis was performed in duplicate.

#### 3.4.7. Antioxidant Activity Determined with ABTS•+

To determine the antioxidant activity against ABTS•+ free radicals, the method described by Re et al. [59] was used. To prepare the cationic solution of the ABTS•+ radical, 0.0384 g of 2,2-azinobis (3-ethylbenzothiazoline-6-sulfonate) cationic radical was weighed into a 10 mL volumetric flask, 0.0066 g of potassium persulfate was added, and then the flask was refilled with distilled water. The solution was mixed and left at room temperature in a dark place for 18 hours. The working solution was obtained by diluting the stock solution with 80% (*v*/*v*) methanol so that the absorbance measured at a wavelength of λ = 734 nm was 0.740–0.750. Up to 0.04 mL of supernatant was obtained following point Section 3.4.1, and 2 mL of ABTS•+ radical working solution was added. The absorbance of the samples was measured after 6 min of incubation at 30 °C at λ = 734 nm. The antioxidant activity against the ABTS•+ radical was calculated using a standard curve prepared for different concentrations (50, 100, 150, 200, 250, and 300 µg/mL) of the ABTS•+ solution and expressed as µM Trolox per 100 g of dry matter (DM) of the sample. The analysis was performed in duplicate.

#### 3.4.8. Sugars Analysis

The sugar was determined in samples of carrots pre-enriched with NFC juices and then dried and raw carrots. The material (approximately 0.5 g), ground in a laboratory grinder (IKA A11 basic; IKA-Werke GmbH, Staufen, Germany), was diluted with 25 mL of distilled water and extracted at 25 °C for 3 h. The obtained solution was centrifuged in a laboratory centrifuge (Rotina 380R, Hettich, Tut-tlingen, Germany) (6500 rpm, 5 min), filtered into vials through a syringe filter with a pore size of 0.45 µm (Macherey-Nagel, Düren, Germany), and dosed into the system. The liquid chromatography method with refractive index (RI) detection was used to determine sugars [60]. A refractive index detector was used—Waters 2414, Milford, MA, USA. Sugar separation was performed using a Waters Sugar-Pak I, 10 µm, 6.5 mm × 300 mm analytical column (Waters, with a Waters Sugar-Pak and Waters Guard-Pak, 10 µm insert). The column was heated to 90 °C and the RI detector to 35 °C. Samples were eluted isostatically using 0.1 mM calcium-disodium EDTA. The injection volume was 10 μL, the mobile phase flow rate was 0.5 mL/min, and the analysis time was 20 min. The quantitative content of analytes was determined based on calibration curves prepared for standard solutions of saccharose, glucose, and fructose. The analysis was performed twice.

### 3.5. Statistical Analysis

The statistical analysis of the obtained results was performed using Microsoft Excel 2016 and STATISTICA 13 PL programs. To determine the effect of pre-treatment, drying method, pressure, and microwave power of microwave-vacuum dryer on selected physicochemical indices, a one- or two-factor analysis of variance was carried out for the mean values from 3 repetitions. Tukey’s HSD test was performed to determine homogeneous groups (post hoc test). Pearson’s correlation was also performed to investigate the relationship between the selected indicators. In addition, principal component (PCA) with classification and cluster analysis was performed.

## 4. Conclusions

The initial thermal treatment of carrots in the form of convective drying, blanching in water, and microwave drying allowed for the reduction of moisture content (increased dry matter content) to a level that allowed for the proper drying of the material using the MVD method or caused physicochemical changes in the tissue enabling MVD drying. For this reason, the use of different pressure and microwave power affects the quality characteristics of the obtained MVD-dried products, which can be achieved without preliminary reduction of the moisture content of the material. 

Osmotic enrichment of carrots in NFC juices, a carrier of biologically active compounds, allowed for obtaining dried products with an increased content of health-promoting ingredients that can be lost during drying using various methods. Even in the case of low-temperature freeze-drying, there was a significant reduction in carotenoids. Enrichment in sea buckthorn juice (SB) allowed for a 41.4 and 46.8% increase in the average carotenoid content compared to the use of pomegranate (PG) and chokeberry (CH) juice and even a nine-fold increase in vitamin C content compared to raw carrot. The use of CH juice had a dominant effect and contributed to a significant increase in TPC content, regardless of the drying method, where the highest value was obtained for FD-dried fruit, even 1255.0 mg GAE/100 g d.m. 

The value of the applied pressure (3.5/6.5 kPa) and microwave power (250/400 W) in MVD drying significantly influenced the technological parameters of microwave-vacuum-dried fruit, such as dry matter content and water activity. These parameters also contributed to changes in the sugar content in the obtained dried fruit. Higher pressure (6.5 kPa) at lower microwave power (250 W) increased total sugar content, regardless of the NFC juice type.

## Figures and Tables

**Table 1:** Dry matter content, water activity, and mass loss in dried carrot; coding markings of dried samples: W_P—without pre-treatment, P-CD—preliminary convective drying (pre-treatment), PG, CH, SB—the type of NFC juices (osmotic enriching pre-treatment), pomegranate, chokeberry, and sea buckthorn juice, respectively; W_BL, M_BL—water blanching, microwave blanching pre-treatment; CD, FD, MVD—convective drying, freeze-drying, microwave-vacuum drying (drying methods); 3.5 or 6.5 kPa_and 250 or 400 W—pressure and microwave power of MVD drying parameters. Within the drying method, the highest and smallest values are marked in bold.

Type of Sample	Dry Matter Content [%]		Water Activity [-]		Mass Loss After Pre-Treatment [%]	Mass Loss After Drying [%]	
Fresh carrot	14.40 ± 0.73		0.987 ± 0.00		-		
CD	87.87 ± 0.53 ^a’ A^		0.404 ± 0.01 ^b’^		-	88.67 ± 0.36 ^B^	
CD_PG	**85.57 ± 0.21** ^a’ AB^		**0.442 ± 0.03** ^b’^		15.75 ± 0.72	**84.00 ± 0.08** ^A^	
CD_CH	89.09 ± 0.03 ^a’ AB^		0.352 ± 0.04 ^b’^		**15.20 ± 0.80**	84.11 ± 0.31 ^A^	
CD_SB	88.64 ± 0.33 ^a’ C^		**0.310 ± 0.01** ^b’^		**9.73 ± 0.61**	88.49 ± 0.11 ^B^	
CD_W_BL	89.50 ± 0.10 ^a’ C^		0.441 ± 0.01 ^b’^		13.99 ± 0.38	**89.80 ± 0.50** ^B^	
CD_M_BL	**91.75 ± 0.34** ^a’ AB^		0.366 ± 0.01 ^b’^		12.34 ± 0.41	93.66 ± 0.50 ^B^	
FD	96.60 ± 0.08 ^b’ A^		0.078 ± 0.01 ^a’^		-	86.21 ± 0.39 ^B^	
FD_PG	**95.25 ± 0.65** ^b’ AB^		0.125 ± 0.02 ^a’^		**6.82 ± 0.26**	82.49 ± 0.22 ^A^	
FD_CH	95.47 ± 0.41 ^b’ AB^		0.128 ± 0.00 ^a’^		**10.09 ± 0.52**	**82.44 ± 0.37** ^A^	
FD_SB	95.87 ± 0.20 ^b’ C^		0.093 ± 0.00 ^a’^		8.95 ± 0.61	85.94 ± 0.68 ^B^	
FD_W_BL	**99.57 ± 0.23** ^b’ C^		**0.070 ± 0.02** ^a’^		8.28 ± 0.18	**90.71 ± 0.50** ^B^	
FD_M_BL	96.18 ± 0.10 ^b’ AB^		**0.135 ± 0.01** ^a’^		8.15 ± 0.03	86.81 ± 0.50 ^B^	
MVD_3.5_250	86.73 ± 0.99 ^a’ A B’^		**0.565 ± 0.01** ^b’ A’^		-	87.20 ± 0.38 ^B^	
MVD_6.5_250	**76.03 ± 0.16** ^a’ A A’^		0.531 ± 0.03 ^b’ B’^		-	**90.78 ± 0.25** ^B^	
MVD_3.5_400	88.00 ± 0.41 ^a’ A A’B’^		0.535 ± 0.01 ^b’ B’^		-	88.47 ± 0.82 ^B^	
MVD_6.5_400	82.39 ± 1.47 ^a’ A A’B’^		0.562 ± 0.01 ^b’ A’B’^		-	87.08 ± 0.32 ^B^	
MVD_3.5_250_PG	**98.57 ± 0.22** ^a’ AB B’^		0.444 ± 0.00 ^b’ A’^		68.43 ± 0.15	85.03 ± 0.28 ^A^	
MVD_6.5_250_PG	91.44 ± 0.40 ^a’ AB A’^		0.380 ± 0.03 ^b’ B’^		65.74 ± 0.22	85.75 ± 0.48 ^A^	
MVD_3.5_400_PG	82.77 ± 2.04 ^a’ AB A’B’^		0.542 ± 0.04 ^b’ B’^		60.75 ± 0.63	84.74 ± 0.39 ^A^	
MVD_6.5_400_PG	94.07 ± 1.60 ^a’ AB A’B’^		0.213 ± 0.01 ^b’ A’B’^		62.84 ± 0.44	84.10 ± 0.35 ^A^	
MVD_3.5_250_CH	92.63 ± 1.07 ^a’ AB B’^		0.343 ± 0.06 ^b’ A’^		55.03 ± 0.83	**84.01 ± 0.11** ^A^	
MVD_6.5_250_CH	81.73 ± 1.73 ^a’ AB A’^		0.527 ± 0.00 ^b’ B’^		51.90 ± 0.55	84.09 ± 0.32 ^A^	
MVD_3.5_400_CH	85.59 ± 1.22 ^a’ AB A’B’^		0.563 ± 0.00 ^b’ B’^		50.96 ± 0.94	84.45 ± 1.40 ^A^	
MVD_6.5_400_CH	93.46 ± 1.37 ^a’ AB A’B’^		0.518 ± 0.00 ^b’ A’B’^		**48.91 ± 0.19**	85.28 ± 0.59 ^A^	
MVD_3.5_250_SB	95.05 ± 0.79 ^a’ C B’^		**0.202 ± 0.01** ^b’ A’^		73.14 ± 0.53	89.67 ± 0.23 ^B^	
MVD_6.5_250_SB	91.19 ± 2.95 ^a’ C A’^		0.425 ± 0.07 ^b’ B’^		70.03 ± 0.75	88.47 ± 0.14 ^B^	
MVD_3.5_400_SB	94.90 ± 0.86 ^a’ C A’B’^		0.254 ± 0.04 ^b’ B’^		69.07 ± 0.04	89.85 ± 0.17 ^B^	
MVD_6.5_400_SB	91.20 ± 0.04 ^a’ C A’B’^		0.367 ± 0.00 ^b’ A’B’^		61.12 ± 1.14	88.59 ± 0.32 ^B^	
MVD_3.5_250_W_BL	95.43 ± 0.10 ^a’ C B’^		0.391 ± 0.00 ^b’ A’^		68.83 ± 0.01	88.73 ± 0.33 ^B^	
MVD_6.5_250_W_BL	91.90 ± 1.31 ^a’ C A’^		0.529 ± 0.05 ^b’ B’^		66.17 ± 0.17	88.44 ± 0.01 ^B^	
MVD_3.5_400_W_BL	94.64 ± 0.17 ^a’ C A’B’^		0.497 ± 0.01 ^b’ B’^		66.95 ± 0.13	90.47 ± 0.51 ^B^	
MVD_6.5_400_W_BL	89.64 ± 0.55 ^a’ C A’B’^		0.455 ± 0.01 ^b’ A’B’^		65.57 ± 0.08	90.13 ± 0.43 ^B^	
MVD_3.5_250_M_BL	92.74 ± 0.65 ^a’ AB B’^		0.304 ± 0.02 ^b’ A’^		71.47 ± 0.48	87.29 ± 0.07 ^B^	
MVD_6.5_250_M_BL	87.37 ± 1.06 ^a’ AB A’^		0.398 ± 0.05 ^b’ B’^		65.46 ± 0.63	86.55 ± 0.10 ^B^	
MVD_3.5_400_M_BL	89.33 ± 0.19 ^a’ AB A’B’^		0.403 ± 0.01 ^b’ B’^		69.36 ± 0.23	88.45 ± 0.86 ^B^	
MVD_6.5_400_M_BL	91.20 ± 0.58 ^a’ AB A’B’^		0.408 ± 0.03 ^b’ A’B’^		**73.81 ± 0.65**	88.83 ± 0.33 ^B^	
MVD_3.5_250_P-CD	88.08 ± 1.42 ^a’ B ’^		0.272 ± 0.03 ^b’ A’^		54.41 ± 0.65	87.02 ± 0.25 ^B^	
MVD_6.5_250_P-CD	94.06 ± 0.70 ^a’ C A’^		0.356 ± 0.04 ^b’ B’^		50.82 ± 1.56	89.39 ± 1.25 ^B^	
MVD_3.5_400_P-CD	92.24 ± 0.05 ^a’ C A’B’^		0.416 ± 0.00 ^b’ B’^		51.12 ± 0.52	87.87 ± 0.14 ^B^	
MVD_6.5_400_P-CD	97.81 ± 0.30 ^a’ C A’B’^		0.283 ± 0.03 ^b’ A’B’^		50.00 ± 0.67	88.85 ± 0.15 ^B^	
	**One-way analysis of variance (ANOVA)**						
**Factors**			**P-probability/Homogenous groups**				
Type of drying method (a’, b’)	0.0001 *	CD ^a’^ FD ^b’^ MVD ^a’^	0.0000 *	CD ^b’^ FD ^a’^ MVD ^b’^	-	0.0538	
Type of pre-treatment (A, B, C)	0.0000 *	W_P ^A^ PG ^AB^ CH ^AB^ SB ^C^ W_BL ^C^ M_BL ^AB^ P-CD ^C^	0.1064		0.8526	0.0000 *	W_P ^B^ PG ^A^ CH ^A^ SB ^B^ W_BL ^B^ M_BL ^B^ P-CD ^B^
	**Two-way analysis of variance (ANOVA)**						
Interaction of MVD drying parameters (A’, B’)	0.0141 *	3.5/250 ^B’^ 6.5/250 ^A’^ 3.5/400 ^A’B’^ 6.5/400 ^A’B’^	0.0147 *	3.5/250 ^A’^ 6.5/250 ^B’^ 3.5/400 ^B’^ 6.5/400 ^A’B’^	-	0.4672	

Different letters a’, b’ (type of drying method); A, B, C (type of pre-treatment) and A’, B’ (pressure and microwave power)—homogeneous groups; show the statistical difference (*—*p* ≤ 0.05). When all the data was in only one homogenous group a’, A, or A’ letters were omitted. The results of mass loss after MVD drying are for the following pre-treatments: blanching (in water (W_BL) or microwave (M_BL)) and preliminary convective drying (P-CD) or osmotic enrichment in pomegranate (PG), chokeberry (CH), and sea buckthorn (SB) juices. Samples CD and FD were not subjected to preliminary convective drying after blanching or osmotic enrichment.

**Table 2:** Pictures of dried carrots, depending on preliminary convection drying (P-CD), water blanching (W_BL), and microwave blanching (M_BL), MVD-drying parameters and drying methods (convection drying (CD), freeze-drying (FD), microwave-vacuum drying (MVD)).

Blanching in Water		Microwave Blanching	
**Convection drying**			
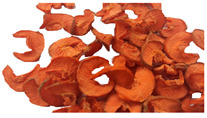		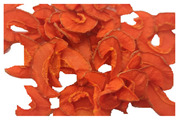	
**Freeze-drying**			
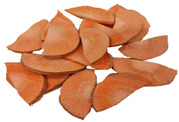		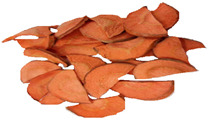	
**Pre-convection drying**	**Blanching in water**		**Microwave blanching**
**Microwave-vacuum drying (3.5 kPa/250 W)**			
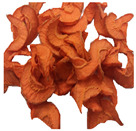	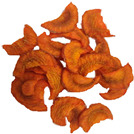		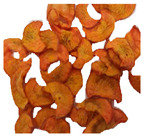
**Microwave-vacuum drying (6.5 kPa/250 W)**			
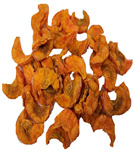	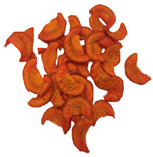		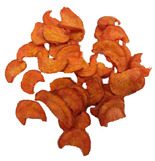
**Microwave-vacuum drying (3.5 kPa/400 W)**			
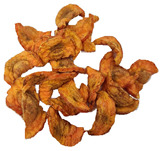	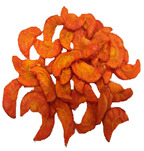		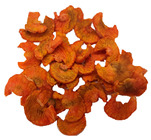
**Microwave-vacuum drying (6.5 kPa/400 W)**			
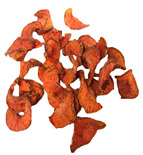	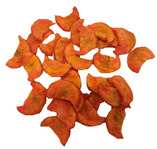		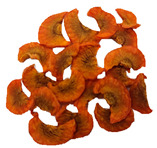

**Table 3:** Influence of the pre-treatment, drying method, and microwave-vacuum-drying parameters on the carotenoid profile in dried carrot; code designations of dried samples are as in the caption of Table 1. Within the drying method, the highest and smallest values are marked in bold.

Type of Samples	Lutein [mg/100 g d.m.]		α-carotene [mg/100 g d.m.]		β-carotene [mg/100 g d.m.]		Total Carotenoids Content [mg/100 g d.m.]	
Fresh carrot	1.00 ± 0.09		10.28 ± 0.21		25.88 ± 0.08		37.16 ± 0.20	
CD	**2.60 ± 0.15** ^c’ A^		**9.23 ± 1.03** ^c’ AB^		21.16 ± 2.65 ^b’ AB^		32.99 ± 3.83 ^b’ AB^	
CD_PG	1.29 ± 0.11 ^c’ A^		**4.57 ± 1.27** ^c’ A^		**8.38 ± 2.23** ^b’ A^		**14.23 ± 3.61** ^b’ A^	
CD_CH	**1.05 ± 0.33** ^c’ A^		5.06 ± 1.35 ^c’ A^		10.80 ± 3.39 ^b’ AB^		16.91 ± 5.07 ^b’ A^	
CD_SB	1.67 ± 0.13 ^c’ A^		8.65 ± 0.10 ^c’ AB^		18.05 ± 0.80 ^b’ AB^		28.37 ± 1.03 ^b’ AB^	
CD_W_BL	1.88 ± 0.01 ^c’ B^		6.09 ± 0.42 ^c’ B^		13.75 ± 0.10 ^b’ B^		21.73 ± 1.43 ^b’ B^	
CD_M_BL	1.68 ± 0.06 ^c’ A^		8.96 ± 1.11 ^c’ AB^		**24.80 ± 2.64** ^b’ AB^		**35.44 ± 3.81** ^b’ AB^	
FD	0.34 ± 0.02 ^a’ A^		**0.76 ± 0.01** ^a’ AB^		**2.03 ± 0.11** ^a’ AB^		**3.20 ± 0.12** ^a’ AB^	
FD_PG	**0.20 ± 0.04** ^a’ A^		1.40 ± 0.35 ^a’ A^		3.27 ± 0.26 ^a’ A^		4.86 ± 0.66 ^a’ A^	
FD_CH	0.30 ± 0.02 ^a’ A^		1.82 ± 0.03 ^a’ A^		4.15 ± 0.20 ^a’ AB^		6.27 ± 0.25 ^a’ A^	
FD_SB	0.37 ± 0.04 ^a’ A^		2.29 ± 0.11^a’ AB^		5.00 ± 0.01 ^a’ AB^		7.65 ± 0.16 ^a’ AB^	
FD_W_BL	**0.76 ± 0.09** ^a’ B^		2.59 ± 0.34 ^a’ B^		7.63 ± 0.72 ^a’ B^		10.98 ± 1.15 ^a’ B^	
FD_M_BL	0.72 ± 0.01 ^a’ A^		**4.40 ± 0.30** ^a’ AB^		**10.80 ± 0.56** ^a’ AB^		**15.92 ± 0.86** ^a’ AB^	
MVD_3.5_250	0.74 ± 0.07 ^b’ A^		2.24 ± 0.51 ^b’ AB^		4.12 ± 0.74 ^a’ AB^		7.11 ± 1.31 ^a’ AB^	
MVD_6.5_250	0.71 ± 0.22 ^b’ A^		4.86 ± 1.82 ^b’ AB^		9.11 ± 3.25 ^a’ AB^		14.68 ± 5.29 ^a’ AB^	
MVD_3.5_400	1.65 ± 0.14 ^b’ A^		5.93 ± 0.16 ^b’ AB^		9.67 ± 0.19 ^a’ AB^		17.25 ± 0.50 ^a’ AB^	
MVD_6.5_400	0.43 ± 0.10 ^b’ A^		2.17 ± 0.33 ^b’ AB^		5.38 ± 0.82 ^a’ AB^		7.99 ± 1.25 ^a’ AB^	
MVD_3.5_250_PG	0.78 ± 0.03 ^b’ A^		4.66 ± 0.47 ^b’ A^		10.94 ± 1.71 ^a’ A^		16.37 ± 2.21 ^a’ A^	
MVD_6.5_250_PG	0.37 ± 0.02 ^b’ A^		1.85 ± 0.01 ^b’ A^		6.20 ± 0.00 ^a’ A^		8.42 ± 0.02 ^a’ A^	
MVD_3.5_400_PG	0.67 ± 0.05 ^b’ A^		2.12 ± 0.15 ^b’ A^		6.50 ± 1.18 ^a’ A^		9.32 ± 1.38 ^a’ A^	
MVD_6.5_400_PG	0.27 ± 0.01 ^b’ A^		**0.55 ± 0.00** ^b’ A^		**1.47 ± 0.12** ^a’ A^		**2.28 ± 0.12** ^a’ A^	
MVD_3.5_250_CH	0.29 ± 0.08 ^b’ A^		2.02 ± 0.27 ^b’ A^		5.69 ± 1.49 ^a’ AB^		8.00 ± 1.85 ^a’ A^	
MVD_6.5_250_CH	0.79 ± 0.24 ^b’ A^		3.74 ± 1.10 ^b’ A^		8.88 ± 2.21 ^a’ AB^		13.40 ± 3.55 ^a’ A^	
MVD_3.5_400_CH	0.65 ± 0.05 ^b’ A^		2.73 ± 0.29 ^b’ A^		7.44 ± 1.27 ^a’ AB^		10.82 ± 1.62 ^a’ A^	
MVD_6.5_400_CH	0.46 ± 0.06 ^b’ A^		1.62 ± 0.05 ^b’ A^		3.68 ± 0.13 ^a’ AB^		5.76 ± 0.14 ^a’ A^	
MVD_3.5_250_SB	**0.24 ± 0.09** ^b’ A^		3.98 ± 0.94 ^b’ AB^		8.54 ± 1.43 ^a’ AB^		12.77 ± 2.46 ^a’ AB^	
MVD_6.5_250_SB	0.47 ± 0.07 ^b’ A^		5.29 ± 0.90 ^b’ AB^		11.06 ± 2.00 ^a’ AB^		16.82 ± 2.98 ^a’ AB^	
MVD_3.5_400_SB	0.57 ± 0.02 ^b’ A^		7.32 ± 0.35 ^b’ AB^		16.08 ± 0.24 ^a’ AB^		23.97 ± 0.57 ^a’ AB^	
MVD_6.5_400_SB	0.51 ± 0.03 ^b’ A^		4.99 ± 0.41 ^b’ AB^		9.30 ± 0.87 ^a’ AB^		14.80 ± 1.31 ^a’ AB^	
MVD_3.5_250_W_BL	1.67 ± 0.01 ^b’ B^		2.66 ± 0.13 ^b’ B^		5.73 ± 0.72 ^a’ B^		25.40 ± 0.74 ^a’ B^	
MVD_6.5_250_W_BL	0.73 ± 0.04 ^b’ B^		2.61 ± 0.06 ^b’ B^		4.31 ± 0.12 ^a’ B^		22.81 ± 7.68 ^a’ B^	
MVD_3.5_400_W_BL	0.85 ± 0.05 ^b’ B^		3.35 ± 0.24 ^b’ B^		7.15 ± 0.03 ^a’ B^		22.23 ± 0.26 ^a’ B^	
MVD_6.5_400_W_BL	1.76 ± 0.01 ^b’ B^		**9.98 ± 1.27** ^b’ B^		**18.66 ± 3.45** ^a’ B^		25.85 ± 3.24 ^a’ B^	
MVD_3.5_250_M_BL	2.02 ± 0.04 ^b’ A^		9.51 ± 0.37 ^b’ AB^		13.87 ± 0.40 ^a’ AB^		10.06 ± 0.87 ^a’ AB^	
MVD_6.5_250_M_BL	2.08 ± 0.54 ^b’ A^		8.67 ± 2.78 ^b’ AB^		12.06 ± 4.37 ^a’ AB^		7.66 ± 0.14 ^a’ AB^	
MVD_3.5_400_M_BL	2.31 ± 0.16 ^b’ A^		6.90 ± 0.05 ^b’ AB^		13.02 ± 0.05 ^a’ AB^		11.35 ± 0.22 ^a’ AB^	
MVD_6.5_400_M_BL	**2.73 ± 0.02** ^b’ A^		8.24 ± 1.06 ^b’ AB^		14.88 ± 2.16 ^a’ AB^		**30.41 ± 4.73** ^a’ AB^	
MVD_3.5_250_P-CD	1.08 ± 0.11 ^b’ A^		4.43 ± 0.44 ^b’ A^		9.76 ± 1.39 ^a’ AB^		15.27 ± 1.94 ^a’ AB^	
MVD_6.5_250_P-CD	0.88 ± 0.02 ^b’ A^		3.86 ± 0.12 ^b’ A^		8.68 ± 0.15 ^a’ AB^		13.42 ± 0.24 ^a’ AB^	
MVD_3.5_400_P-CD	1.34 ± 0.15 ^b’ A^		3.42 ± 0.34 ^b’ A^		7.75 ± 0.73 ^a’ AB^		12.51 ± 1.22 ^a’ AB^	
MVD_6.5_400_P-CD	0.92 ± 0.16 ^b’ A^		2.05 ± 0.54 ^b’ A^		4.51 ± 0.54 ^a’ AB^		7.49 ± 1.24 ^a’ AB^	
**One-way analysis of variance (ANOVA)**								
**Factors**	**P-probability/Homogenous groups**							
Type of drying method (a’, b’, c’)	0.0000 *	CD ^c’^ FD ^a’^ MVD ^b’^	0.0000 *	CD ^c’^ FD ^a’^ MVD ^b’^	0.0000 *	CD ^c’^ FD ^a’^ MVD ^b’^	0.0001 *	CD ^c’^ FD ^a’^ MVD ^b’^
**Two-way analysis of variance (ANOVA)**								
Type of pre-treatment (A, B)	0.0000 *	W_P ^A^ PG ^A^ CH ^A^ SB ^A^ W_BL ^B^ M_BL ^A^ P-CD ^A^	0.0001 *	W_P ^AB^ PG ^A^ CH ^A^ SB ^AB^ W_BL ^B^ M_BL ^AB^ P-CD ^A^	0.0086 *	W_P ^AB^ PG ^A^ CH ^AB^ SB ^AB^ W_BL ^B^ M_BL ^AB^ P-CD ^AB^	0.0014 *	W_P ^AB^ PG ^A^ CH ^A^ SB ^AB^ W_BL ^B^ M_BL ^AB^ P-CD ^AB^
Interaction of MVD drying parameters (A’, B’, C’)	0.9508		0.7296		0.4922		0.5932	

Different letters a’, b’ c’ (type of drying method); A, B (type of pre-treatment), and A’, B’, C’ (pressure and microwave power)—homogeneous groups; show the statistical difference (*—*p* < 0.05). When all the data were in only one homogenous group, a’, A, or A’ letters were omitted.

**Table 4:** Influence of the pre-treatment, drying method, and microwave-vacuum-drying parameters on the anthocyanins profile in dried carrot snacks; code designations of dried samples are as in the caption of Table 1. The highest values are marked in bold.

Type of Samples	Cy-3-O-gal [mg/100 g d.m.]	Cy-3-O-glu [mg/100 g d.m.]	Cy-3-O-ara [mg/100 g d.m.]	Cy-3-O-xyl [mg/100 g d.m.]	Total Anthocyanins [mg/100 g d.m.]
CD_CH	1.28 ± 0.27 ^b’^	0.10 ± 0.01 ^b’^	0.26 ± 0.12 ^b’^	2.67 ± 0.35 ^a’^	4.30 ± 0.63 ^b’^
FD_CH	**12.61 ± 0.43** ^c’^	**0.74 ± 0.04** ^c’^	**5.15 ± 0.17** ^c’^	**4.07 ± 0.18** ^b’^	**22.57 ± 0.79** ^c’^
MVD_3.5_250_CH	0.31 ± 0.05 ^a’ B’^	0.05 ± 0.00 ^a’^	0.10 ± 0.05 ^a’^	2.78 ± 0.15 ^a’ B’^	3.24 ± 0.21 ^a’ B’^
MVD_3.5_400_CH	0.30 ± 0.02 ^a’ B’^	0.02 ± 0.00 ^a’^	0.09 ± 0.02 ^a’^	2.25 ± 0.38 ^a’ A’B’^	2.67 ± 0.37 ^a’ A’B’^
MVD_6.5_250_CH	0.23 ± 0.04 ^a’ A’^	0.04 ± 0.02 ^a’^	0.09 ± 0.02 ^a’^	2.03 ± 0.10 ^a’ A’^	2.40 ± 0.10 ^a’ A’^
MVD_6.5_400_CH	0.26 ± 0.03 ^a’ A’B’^	0.03 ± 0.02 ^a’^	0.07 ± 0.03 ^a’^	2.38 ± 0.22 ^a’ A’B’^	2.75 ± 0.23 ^a’ A’B’^

Different letters a’, b’, c’ (type of drying method); A’, B’ (pressure and microwave power)—homogeneous groups show the statistical difference (*p* < 0.05). When all the data were in only one homogenous group, a’ or A’ letters were omitted.

**Table 5:** Influence of the pre-treatment, drying method, and microwave-vacuum-drying parameters on the L-ascorbic acid (AA), L-dehydroascorbic acid (DHAA), and total vitamin C content of dried carrot; code designations of dried samples are as in the caption of Table 1. Within the drying method, the highest and smallest values are marked in bold.

Type of Samples	AA [mg/100 g d.m.]	DHAA [mg/100 g d.m.]	Total Vitamin C (AA + DHAA) [mg/100 g d.m.]
Fresh carrot	0.06 ± 0.01 ^A^	4.83 ± 0.04 ^a’b’ BC^	4.89 ± 0.04 ^a’ A^
CD_PG	4.77 ± 0.17 ^A^	1.04 ± 0.63 ^a’ A^	5.81 ± 0.46 ^c’ A^
CD_CH	**0.92 ± 0.08** ^A^	3.43 ± 0.11 ^a’ AB^	**4.34 ± 0.03** ^c’ A^
CD_SB	**41.15 ± 0.36** ^B^	1.46 ± 0.11 ^a’ C^	**42.62 ± 0.25** ^c’ B^
FD_PG	0.02 ± 0.00 ^A^	2.35 ± 0.04 ^b’ A^	**2.37 ± 0.03** ^a’ A^
FD_CH	0.05 ± 0.02 ^A^	4.45 ± 0.03 ^b’ AB^	4.40 ± 0.01 ^a’ A^
FD_SB	**10.33 ± 0.40** ^B^	**7.41 ± 0.04** ^b’ C^	**17.74 ± 0.37** ^a’ B^
MVD_3.5_250_PG	0.87 ± 0.02 ^A^	**0.19 ± 0.02** ^a’ A^	1.06 ± 0.00 ^b’ A^
MVD_6.5_250_PG	0.48 ± 0.01 ^A^	0.51 ± 0.04 ^a’ A^	**0.99 ± 0.05** ^b’ A^
MVD_3.5_400_PG	0.84 ± 0.00 ^A^	0.78 ± 0.05 ^a’ A^	1.62 ± 0.04 ^b’ A^
CMVD_6.5_400_PG	0.14 ± 0.05 ^A^	1.06 ± 0.04 ^a’ A^	1.20 ± 0.01 ^b’ A^
MVD_3.5_250_CH	0.45 ± 0.01 ^A^	1.26 ± 0.30 ^a’ AB^	1.72 ± 0.29 ^b’ A^
MVD_6.5_250_CH	0.39 ± 0.08 ^A^	1.05 ± 0.10 ^a’ AB^	1.44 ± 0.02 ^b’ A^
MVD_3.5_400_CH	0.27 ± 0.00 ^A^	1.59 ± 0.03 ^a’ AB^	1.86 ± 0.02 ^b’ A^
MVD_6.5_400_CH	0.41 ± 0.02 ^A^	1.40 ± 0.06 ^a’ AB^	1.81 ± 0.04 ^b’ A^
MVD_3.5_250_SB	35.29 ± 0.64 ^B^	3.00 ± 1.86 ^a’ C^	38.29 ± 1.23 ^b’ B^
MVD_6.5_250_SB	36.23 ± 1.49 ^B^	4.70 ± 0.32 ^a’ C^	40.93 ± 1.17 ^b’ B^
MVD_3.5_400_SB	31.50 ± 0.44 ^B^	4.20 ± 0.35 ^a’ C^	35.70 ± 0.79 ^b’ B^
MVD_6.5_400_SB	**40.65 ± 0.17** ^B^	4.32 ± 0.56 ^a’ C^	**44.97 ± 0.39** ^b’ B^

Different letters a’, b’ (type of drying method); A, B, C (type of NFC juice)—homogeneous groups show the statistical difference (*p* < 0.05). When all the data were in only one homogenous group, a’, A letters were omitted.

**Table 6:** Characteristics of NFC juices used for osmotic enrichment.

NFC Juice	pH [-]	Water Activity [-]		Color Parameters	
			L*	a*	b*
Pomegranate (PG)	3.23 ± 0.01 ^b^	0.974 ± 0.002 ^b^	19.16 ± 0.07 ^b^	10.26 ± 0.1 ^b^	4.06 ± 0.08 ^b^
Chokeberry (CH)	3.60 ± 0.01 ^b^	0.971 ± 0.001 ^a^	1.40 ± 0.08 ^a^	−0.02 ± 0.15 ^a^	−0.25 ± 0.14 ^a^
Sea buckthorn (SB)	2.77 ± 0.01 ^a^	0.985 ± 0.001 ^c^	44.18 ± 0.03 ^c^	23.33 ± 0.04 ^c^	53.04 ± 0.12 ^c^

Different letters a, b, c (type of NFC juice) in columns show the statistical difference (*p* < 0.05). Color Parameters L*, a*, b*—lightness, redness, yellowness.

**Table 7:** Sugar content in NFC juices used for osmotic enrichment.

Sugar Content [g/L]				
NFC Juice	Saccharose	Glucose	Fructose	Sorbitol
Pomegranate (PG)	4.47 ± 0.07 ^b^	60.62 ± 0.29 ^c^	68.00 ± 0.06 ^c^	≤150 mg/L #
Chokeberry (CH)	<1 ^a^	34.56 ± 0.27 ^b^	21.59 ± 0.48 ^b^	43.95 ± 0.59
Sea buckthorn (SB)	14.58 ± 0.50 ^c^	10.17 ± 0.03 ^a^	2.88 ± 0.16 ^a^	≤150 mg/L #

Different letters a, b, c (type of NFC juice) in columns show the statistical difference (*p* < 0.05); #—below the limit of quantification (LOQ).

**Table 8:** Parameters of microwave-vacuum drying (MVD).

Parameters/ Cycles	Cycle I	Cycle II	Cycle III	Cycle IV Stabilization
**Pressure (kPa)**	3.5/6.5	3.5/6.5	3.5/6.5	-
**Microwave power (W)**	250/400	-	250/400	-
**Temperature (°C)**	70	-	70	-
**Time (s)**	120–630	210	120–630	210

## Data Availability

The data presented in this study are available on request from the corresponding author.

## Supplementary Materials

The following supporting information can be downloaded at: https://www.mdpi.com/article/10.3390/molecules29215100/s1, Table S1: Comparison of color parameters and the absolute color difference of drying carrots, Table S2: Pictures of dried carrots, depending on NFC juice used for osmotic enrichment and drying methods, Figure S1: PCA Scree plot of data: (a) in terms of technological properties related to thermal pretreatment (blanching and convective drying) and (b) in terms of properties of dried carrots after pre-enrichment.
