# Supplementary material for: Shaping the Physicochemical and Health-Promoting Properties of Carrot Snacks Produced by Microwave-Vacuum Drying with Preliminary Thermal and Enriching Treatment
Source: Molecules. 2024 Oct 29;29(21):5100. doi: 10.3390/molecules29215100 (PMC11547784; doi:10.3390/molecules29215100)
Supplement: Supplementary file 1 [file molecules-29-05100-s001.zip › molecules-3212910-supplementary.pdf]

Supplementary Materials:

Shaping the Physicochemical and Health-Promoting Properties of Carrot Snacks Produced by Microwave-Vacuum Drying with Preliminary Thermal and Enriching Treatment

Table S1. Comparison of color parameters L*, a*, b*, ΔE- lightness, redness, yellowness, and absolute color difference of drying carrots; coding markings of dried samples: W_P (without pre-treatment), P-CD – preliminary convective drying (pre-treatment), PG, CH, SB – the type of NFC juices (osmotic enriching pre-treatment), pomegranate, chokeberry, and sea buckthorn juice, respectively; W_BL, M_BL – water blanching, microwave blanching pre-treatment; CD, FD, MVD – convective drying, freeze-dying, microwave-vacuum drying (drying methods); 3.5 or 6.5 kPa and 250 or 400 W– pressure and microwave power of MVD drying parameters.

Type of samples	Color Parameters			
	L*	a*	b*	ΔE
Fresh carrot	65.37 ± 0.8	25.58 ± 1.49	36.83 ± 1.14	-
CD	**62.10 ± 6.06**	28.28 ± 1.87	38.42 ± 2.10	**6.42 ± 3.12**
CD_PG	38.60 ± 2.75	22.62 ± 2.17	21.86 ± 2.07	29.01 ± 3.64
CD_CH	**18.80 ± 1.32**	**15.60 ± 1.40**	**3.20 ± 0.55**	**57.03 ± 1.57**
CD_SB	46.47 ± 0.99	**32.77 ± 1.94**	**46.94 ± 1.05**	16.11 ± 0.58
CD_W_BL	49.52 ± 3.64	30.28 ± 2.47	32.41 ± 2.16	13.62 ± 3.97
CD_M_BL	49.87 ± 1.78	27.66 ± 5.14	32.79 ± 3.03	13.76 ± 3.45
FD	71.20 ± 2.06	27.65 ± 2.03	42.74 ± 1.15	11.56 ± 1.93
FD_PG	55.53 ± 0.73	**28.96 ± 0.82**	26.05 ± 1.77	14.86 ± 1.81
FD_CH	**25.76 ± 1.92**	25.62 ± 1.65	**5.89 ± 0.75**	**48.72 ± 1.78**
FD_SB	65.18 ± 0.98	28.94 ± 0.30	**58.06 ± 2.31**	18.65 ± 2.36
FD_W_BL	**74.26 ± 2.48**	**18.58 ± 1.99**	37.28 ± 5.17	18.13 ± 3.70
FD_M_BL	68.56 ± 4.21	23.08 ± 2.50	37.42 ± 1.87	**11.43 ± 1.95**
MVD_3.5_250	62.73 ± 2.61	5.05 ± 2.03	9.86 ± 3.23	38.55 ± 3.56
MVD_6.5_250	59.55 ± 1.28	**3.55 ± 0.72**	6.74 ± 1.81	41.77 ± 1.89
MVD_3.5_400	62.63 ± 1.73	5.20 ± 1.09	10.07 ± 2.11	38.22 ± 2.17
MVD_6.5_400	34.56 ± 4.75	10.83 ± 2.67	23.98 ± 6.08	35.49 ± 6.68
MVD_3.5_250_PG	33.52 ± 1.6	9.64 ± 0.74	16.53 ± 0.63	40.42 ± 1.08
MVD_6.5_250_PG	40.40 ± 2.42	15.53 ± 2.38	21.98 ± 3.59	30.16 ± 3.07
MVD_3.5_400_PG	41.85 ± 1.99	18.55 ± 2.14	21.99 ± 1.75	27.87 ± 1.62
MVD_6.5_400_PG	38.33 ± 3.57	13.90 ± 1.41	22.06 ± 1.53	32.07 ± 3.49
MVD_3.5_250_CH	23.81 ± 2.27	12.62 ± 2.09	5.29 ± 0.67	52.93 ± 2.34
MVD_6.5_250_CH	26.43 ± 2.03	14.81 ± 1.60	6.04 ± 0.83	49.98 ± 2.20
MVD_3.5_400_CH	27.05 ± 0.45	16.45 ± 1.17	5.94 ± 0.49	49.19 ± 0.80
MVD_6.5_400_CH	**23.78 ± 0.93**	13.28 ± 1.44	**4.54 ± 0.79**	**53.22 ± 1.44**
MVD_3.5_250_SB	37.69 ± 3.64	15.08 ± 1.02	31.71 ± 3.02	27.79 ± 3.90
MVD_6.5_250_SB	51.29 ± 2.81	24.37 ± 3.69	49.88 ± 2.73	14.92 ± 0.92
MVD_3.5_400_SB	50.40 ± 3.20	21.96 ± 4.07	49.15 ± 5.54	16.71 ± 1.47
MVD_6.5_400_SB	51.39 ± 1.37	22.29 ± 2.39	**50.22 ± 2.82**	15.36 ± 1.02
MVD_3.5_250_W_BL	51.54 ± 2.98	23.22 ± 1.96	49.96 ± 3.71	14.82 ± 3.12
MVD_6.5_250_W_BL	50.82 ± 1.61	24.55 ± 0.90	49.89 ± 2.59	14.44 ± 1.85
MVD_3.5_400_W_BL	49.12 ± 2.81	20.51 ± 2.09	42.86 ± 6.34	15.74 ± 1.59
MVD_6.5_400_W_BL	54.30 ± 3.79	19.18 ± 2.69	46.04 ± 4.83	14.08 ± 2.64
MVD_3.5_250_M_BL	**57.98 ± 1.63**	17.18 ± 2.21	48.39 ± 4.09	15.04 ± 1.78
MVD_6.5_250_M_BL	56.87 ± 2.48	23.17 ± 2.16	50.02 ± 3.60	12.44 ± 2.96

MVD_3.5_400_M_BL	55.60 ± 3.07	**26.77 ± 2.96**	39.43 ± 3.01	**6.98 ± 2.07**
MVD_6.5_400_M_BL	54.96 ± 2.69	26.53 ± 1.79	49.45 ± 3.97	11.89 ± 2.19
MVD_3.5_250_P-CD	54.06 ± 4.50	11.47 ± 2.05	28.01 ± 2.19	22.39 ± 2.67
MVD_6.5_250_P-CD	57.32 ± 3.40	14.31 ± 4.15	32.82 ± 5.47	17.25 ± 5.30
MVD_3.5_400_P-CD	53.67 ± 5.96	11.48 ± 1.27	36.85 ± 6.99	20.22 ± 5.04
MVD_6.5_400_P-CD	41.09 ± 2.26	8.17 ± 0.86	22.97 ± 1.46	32.94 ± 1.79

One-way analysis of variance (ANOVA)

**Factors**	**P-probability/ Homogenous groups**							
Type of drying method (a′, b′)	0.0001*	CD $^{a'}$<br>FD $^{a'}$<br>MVD $^{a'}$	0.0001*	CD $^{b'}$<br>FD $^{b'}$<br>MVD $^{a'}$	0.3294		0.0382*	CD $^{a'b'}$<br>FD $^{a'}$<br>MVD $^{b'}$
Type of pre-treatment (A, B, C, D)	0.0001*	W_P<br>PG ^{B}<br>CH ^{A}<br>SB ^{C}<br>W_BL ^{CDE}<br>M_BL ^{DE}<br>P-CD ^{CD}	0.0000*	W_P<br>PG ^{BC}<br>CH ^{AB}<br>SB ^{D}<br>W_BL ^{CD}<br>M_BL ^{D}<br>P-CD ^{A}	0.0001*	PG ^{B}<br>CH ^{A}<br>SB ^{D}<br>W_BL ^{D}<br>M_BL ^{D}<br>CD ^{C}	0.0001*	W_P<br>PG ^{D}<br>CH ^{E}<br>SB ^{BC}<br>W_BL ^{AB}<br>M_BL ^{A}<br>P-CD ^{CD}

Two-way analysis of variance (ANOVA)

Interaction of MVD drying parameters (A', B', C')	0.0874	0.1303	0.9388	0.1770

*—means significant difference at a confidence level of 0.05; different letters a′, b′; A, B, C, D and A′, B′, C′ – homogenous groups. When all the data was in one homogenous group ($p > 0.05$) a′, A or A′ letters were omitted.

Table S2. Pictures of dried carrots, depending on NFC juice used for osmotic enrichment and drying methods; coding markings of dried samples: P-CD–convective drying (pre-treatment), PG, CH, SB–the type of NFC juices (osmotic enriching pre-treatment), pomegranate, chokeberry, and sea buckthorn juice, respectively; W_BL, M_BL–water blanching, microwave blanching pretreatment; CD, FD, MVD–convective drying, freeze-dying, microwave-vacuum drying (drying methods); 3.5 or 6.5 kPa and 250 or 400 W– pressure and microwave power of MVD drying parameters..

**Without pre-treatment**	**Pre-osmotic enrichment in PG juice**	**Pre-osmotic enrichment in CH juice**	**Pre-osmotic enrichment in SB juice**

Convection drying

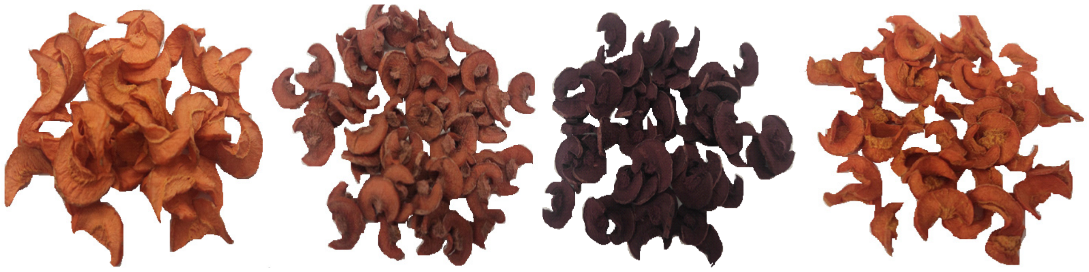

Freeze-drying

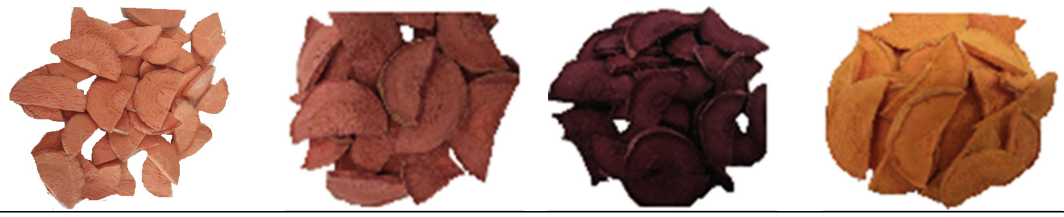

Microwave-vacuum drying (3.5 kPa / 250 W)

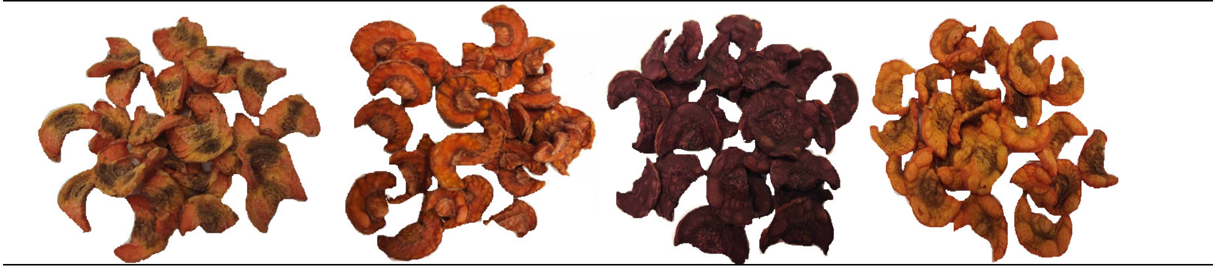

Microwave-vacuum drying (6.5 kPa / 250 W)

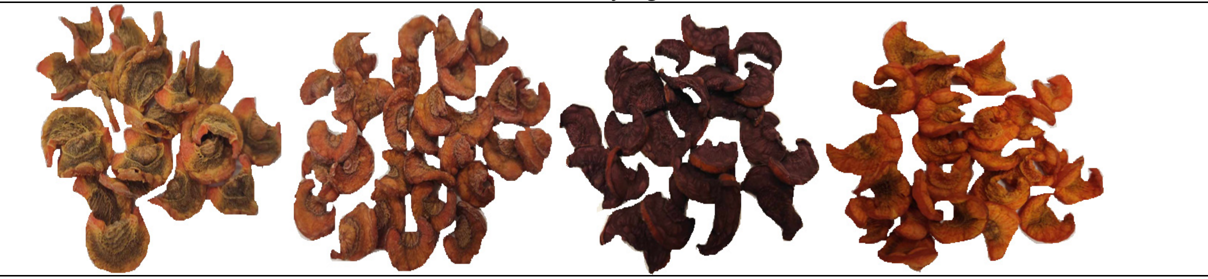

Microwave-vacuum drying (3.5 kPa / 400 W)

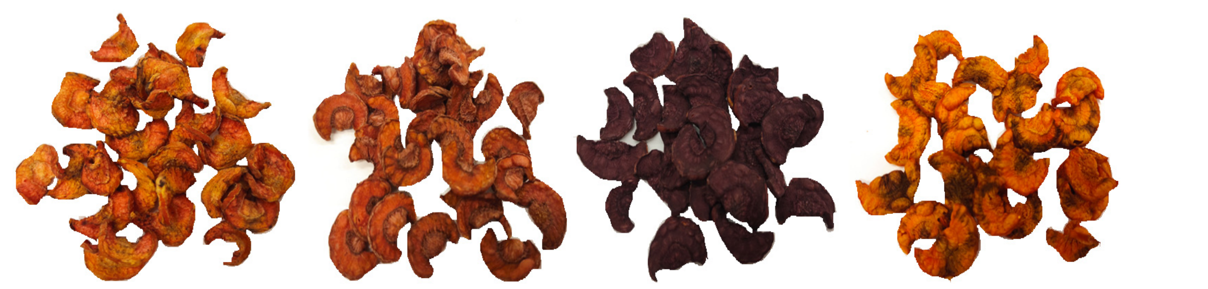

Microwave-vacuum drying (6.5 kPa / 400 W)

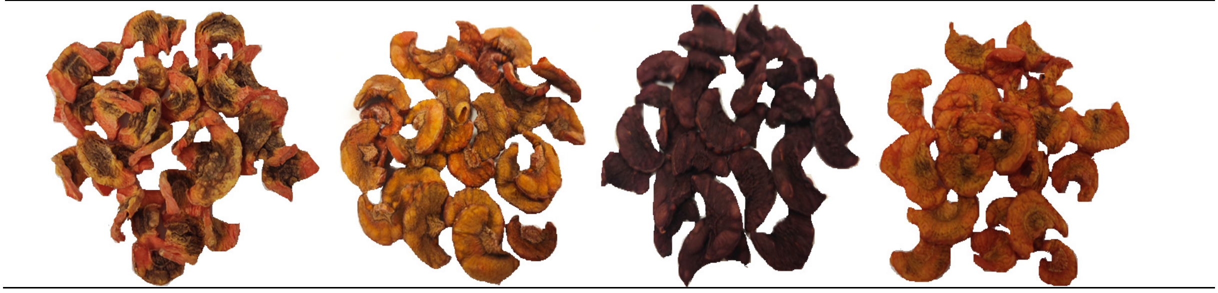

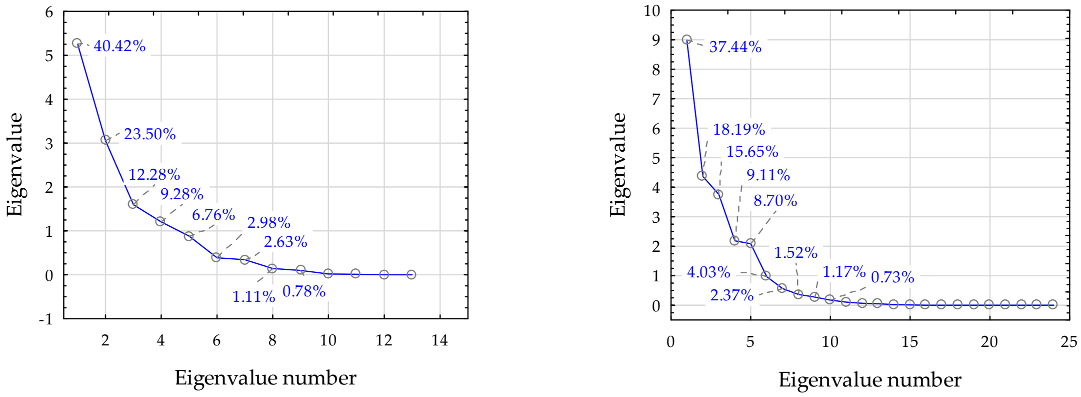

(a) (b)

Figure S1. PCA Scree plot of data: (a) in terms of technological properties related to thermal pretreatment (blanching and convective drying) and (b) in terms of properties of dried carrots after pre-enrichment.
